# Macro-micro dynamics of child undernutrition in Pakistan: a structural learning-based Bayesian network analysis of household and policy drivers

**DOI:** 10.3389/fnut.2026.1882524

**Published:** 2026-10-06

**Authors:** Muhammad Shahid, Jiayi Song, Zaiba Ali, Hafiz Muhammad Naveed, Serhat Yuksel, Hasan Dincer

**Affiliations:** 1 College of Management, Shenzhen University, Shenzhen, Guangdong, China; 2 Department of Family Medicine, Faculty of Medicine and Health Sciences, McGill University, Montreal, QC, Canada; 3 Department of Management, College of Business Administration, Princess Nourah bint Abdulrahman University, Riyadh, Saudi Arabia; 4 School of Management Science and Engineering, Shandong University of Finance and Economics, Shandong, Jinan, China; 5 School of Business, Istanbul Medipol University, Istanbul, Türkiye; 6 Clinic of Economics, Azerbaijan State University of Economics (UNEC), Baku, Azerbaijan; 7 Department of Economics and Management, Khazar University, Baku, Azerbaijan; 8 University College, Korea University, Seoul, Republic of Korea

**Keywords:** child undernutrition, inequality, macro-micro determinants, nutrition policies and governance, Pakistan, SL-BN

## Abstract

**Background:**

Child undernutrition remains a major public health concern and creates health-related inequalities in developing countries, with long-term consequences for human development deficits. Most of these early studies were conducted using conventional econometric techniques; however, understanding the complex, multi-level determinants of undernutrition may require more advanced analytical methods.

**Methods:**

This study, PC algorithm is used for Structural Learning-Based Bayesian Networks [SL-BN] (a graphical model based on directed acyclic graph with joint and conditional associations)—which is conceptually different than the weight-based networks, to examine the macro- and micro-level determinants of child undernutrition in Pakistan. Macro-level analysis combines historical nutrition policy evaluation (critical discourse) with WDI and UNDP data (2000–2022), spanning the MDG and SDG eras. Micro-level analysis draws on the Pakistan Demographic and Health Survey (PDHS 2017–18) to identify socioeconomic drivers of child malnutrition and nutritional inequalities.

**Results:**

In micro analysis, the SL-BN network structure identifies household-level determinants—such as poor wealth status, rural residence, poor environmental conditions, low maternal education, and diarrhea—as key drivers of child undernutrition. Inequality is stark, with malnutrition disproportionately concentrated among the poorest. However, the SL-BN findings and critical assessment of nutritional policies and programs found that low investment in human development, foreign dependency on health, poverty, inflation, and a history of inconsistent nutritional policies to combat hunger and undernutrition are the fundamental issues at the macro level in Pakistan.

**Conclusion:**

The study concluded that the underlying micro level determinants of child undernutrition in Pakistan cannot overcome without long-term targeted policies at macro level.

## Highlights

- Micro and macro factors substantially impact child malnutrition in Pakistan.
- Micro-level undernutrition is more concentrated among disadvantaged groups of wealth status.
- Income and government/private spending conditionally improve child malnutrition by given the percentage contribution of human development index at macro-level.
- Higher mother education, BMI, and water and sanitation reduce the likelihood of child undernutrition by given the percentage effect of household wealth status at the micro-level.
- The Bayesian network is suitable for analyzing joint probability distributions and conditional dependencies among random variables in network structure.

## Introduction

Developing countries bear the brunt of child malnutrition, which is a major issue in public health worldwide. It accounts for approximately fifty percent of all annual child fatalities, with one in three child deaths associated with severe acute malnutrition (1). South Asia carries the greatest burden, housing almost 57.9 million malnourished children—two out of every five worldwide (2). Nearly 38% of children below five years in India and Pakistan together have the highest stunting rates among all countries (3).

Pakistan, despite being the sixth most populous country in the world with a population of over 220 million, has been one of the major challenges for Pakistan though since 2015 around 24.3% of people lived below the income poverty line which improved to 21.9% till year 2018. Moreover, more than 38% of the population is experiencing multi-dimensional poverty where considerable deprivations exist in health status, education status, and living standards with strong variations across regions in the country (4, 5). These socioeconomic adversities are reflected grimly in child nutrition outcomes: 38.1% (or 8 million) children under five years are stunted, 23.0% (or 4.5 million) children suffer from underweight, and wasting affects 8.1% (6). Pakistan has wider income and poverty gaps than its neighboring countries, which affects the distribution of nutrition (7). The prevalence of malnutrition is up to 17% greater in affluent toward lower socioeconomic households (8, 9). The data continues to show that the effect of malnourishment is disproportionately worse among poorer and rural people than wealthier urban ones.

The prevalence of under-nutrition is highest in provinces like Sindh and Balochistan where the highest rates of stunting (47.4% and 49.3%) are recorded. Such numbers would be a stark departure from what has been reported in Punjab, Khyber Pakhtunkhwa (KPK), and the Islamabad Capital Territory [ICT] (National Institute of Population Studies, 2018). Socioeconomic inequality compounds the problem; for example, malnourishment affects 56% of children from poor homes, whereas only 22% of those in financially secure households go hungry. These regional and economic disparities are determinant of dietary and nutritional outcome in low- and middle-income countries, where adequate nutrition holds significant importance for child development (10, 11). Poor diets are often a marker of wider structural inequalities in society, and low income has long been established as the major influence on undernutrition (12, 13).

The role of child malnutrition as an indicator of poverty is obvious; it not only indicates current periods of poverty but also a predictor of economic adversity in adulthood due to decreased productivity and earning ability (9). Poor feeding practices, unavailability of clean water and inadequate WASH are prevalent in poor households in Pakistan that significantly contribute to the risk of undernutrition (9, 14). This scenario is ascribed to several enabling factors, amongst which are low levels of maternal education (15), inadequate maternal health and nutrition (16), as well as long-term unemployment of the head of the household (17), both key determinants for healthy child development. Economically, malnutrition has dire consequences with a reported loss of Pakistan's annual GDP deducted up to 3% (Pakistan Economic Survey, 2018–19). Specifically, stunting incurs productivity losses of 6–8.6% in agriculture and manufacturing sectors, and up to 19.8% in services, resulting in an annual economic loss of almost $2 billion (18).

Research in developing countries has shown that socioeconomic factors—both affecting individuals separately or rippling together—have a large impact on child nutrition outcomes (19). The main factors are poverty, inflation, illiteracy of mother, low intake of food, unavailability of clean water and sanitation facilities and regional imbalance (20–25). Critics contend that the biomedical and specifically developmental frameworks—which largely center on the “individual body”—fail to attend to more expansive socio-political and economic contexts of hunger and human suffering (26, 27). The focus of political economists, to name a few, is on the structural problems which include ineffective nutrition policies in Pakistan's colonial past, stagnant and stagnant poverty levels, inflationary pressures (27), reliance on foreign debt and broken governance of indicators such as undernutrition in Pakistan (27, 28).

Undernutrition among children is determined by the interplay of micro-level (underlying) and macro-level (fundamental) factors. On the micro level, the individual or household may be facing poor nutritional outcomes due to a lack of socio-economic capital that is impacted by challenges posed at macro level around structural and policy related issues. Most prior studies examining child undernutrition in Pakistan have relied on conventional econometric approaches, including logistic regression (14, 20), multilevel regression models (15), and decomposition analysis (9). These methods have provided valuable information, though they are limited by important assumptions: (1) the logistic regression relies on linearity, additivity and independence among predictors—assumptions that are often violated in multidimensional malnutrition determinant contexts; (2) multilevel models can partially address hierarchical data structures but do not fully account for complex conditional dependencies between exposures along with non-linear interactions of socioeconomic, environmental and maternal factors; (3) traditional methods largely estimate marginal effects rather than jointly modeling probabilistic relationships combined with conditional independencies to characterize causal pathways of undernutrition. And these methodological constraints have left a most canyons in our understanding of how structural and household determinants interact to influence child nutritional outcomes. To tackle these limitations, this study utilizes a Bayesian network model—a type of probabilistic graphical model that allows for appropriate representation of complex conditional dependencies, non-linear relationships between variables, and interpretable DAG explicit communication of the structural relationships among variables. This is the first study that adopts Bayesian networks to analyze the macro and micro factors contributing to child undernutrition in Pakistan. This research has applied the analytical framework of Bayesian networks to decompose all possible factors leading to child undernutrition in Pakistan, categorize them into macro and micro levels. For transparency on to ensure scientific novelty, this study's analysis is advanced on the basis of the relevant research previously done using same PDHS 2017–18 datasets (20–22, 112, 121). Compared with the previous work, this analysis adds three new research contributions that have never been observed before:

(1) Adopt the SL-BN method to analyses the conditional dependence relationships between different variables;
(2) Integrate various relevant factors at the micro household level and various relevant factors at the macro policy level to carry out the analysis;
(3) Combine the quantitative inequality measurement method, that is, the Concentration Index, with qualitative policy discussions to conduct triangulation. The descriptive statistics and logistic regression used in this study only serve one purpose, which is to test whether the results obtained from the Bayesian network analysis are reliable, that is, they are only used as a robustness test, and these two methods will not be used to produce core research conclusions.

In section 2 we present the research setting (the theoretical basis and key independent variables for consideration) as well as the model equations used in each regression of interest, alongside our robustness checks. The current section gives a comprehensive description of the empirical usage of these models through their role in addressing the complex challenge that under-nutrition, and children under-nutrition especially represents. The final section consists of two sub-sections analyzing the results micro (socioeconomic inequity vs. rural inequality in calories consumption per day by wealth quantiles in Pakistan) level and macro (nutrition policy)-level variables. Section 4 presents the main results of the study, followed by policy implications and limitations in Section 5, and a conclusion.

## Data and methodology

### Data, study designing, and analytical sample flow

In this study, we assumed that the drivers of child undernutrition in Pakistan are combinations of micro- and macro-level determinants, pointed out the root causes using a mixed-methods approach to explain these primal factors contributing to this overall public health challenge. These included: a systematic review of the evolution of vitamin supplementation, nutrition policies, programmes and initiatives from a health system perspective at national level; an assessment of their sustainability to support primary interventions. The framework of analysis was based on an analytical framework built on time-series datasets covering key macroeconomic and human development indicators from 2000 to 2022. Long-term structural drivers of child nutrition were obtained from comprehensive high-quality databases such as the World Development Indicators (WDI) and the UNDP.

The sampling design of the PDHS was a mega multi-stage stratified cluster sampling approach, which had features to ensure national representation. Primary sampling units (PSUs) were sampled using probability proportional to size within each stratum and households were selected at random within each PSU. To account for this complex survey design, all descriptive statistics, logistic regression analyses, and concentration index calculations incorporated appropriate sampling weights (v005 variable in PDHS) to produce nationally representative estimates. For the Bayesian network analysis, which is based on conditional independence relationships rather than population-level prevalence estimates, sampling weights were not applied following standard practice for structural learning in Bayesian networks (Scutari & Denis, 2014)^1^. However, for the logistic regression and concentration index analyses, we used the survey set commands in STATA (svyset) specifying the primary sampling unit (PSU), stratum, and sampling weights to ensure correct variance estimation and population representativeness. The PDHS data provide a wide-ranging definition of maternal and child health in the country along with a more focused presentation of nutritional status as well as demographic, socioeconomic and other characteristics of these populations. We assessed child growth through standardized anthropometric measurements and appropriate statistical protocols were used to evaluate the data. We established four classes of clinical determinates which were class one (socioeconomic characteristics), class two, (maternal and child variables), class three (disease incidence) and, finally, class Four dietary habits. Table 1 lists the data and variables used in this study.

**Table 1:** Variables, operationalization, and data sources.

PART A: Micro-level variables (Pakistan demographic and health survey, 2017–18)			
Variable category	Variable	Operational definition	Source
15.6-7.4,-13.8498ptDependent Variable	Child malnutrition (CIAF)	Dummy variable: 1 = malnourished, 0 = otherwise.	PDHS
Child-level characteristics			
Sex		1 = male, 0 = female.	PDHS
Age (months)		1 = 0–12, 2 = 13–24, 3 = 25–36, 4 = 37–48, 5 = 49–60.	PDHS
Birth interval (time since previous birth)		“1” value considered if ≤ 2 years since previous birth, “2” value given if the order is 3–4 years, “3” value given if the order is 5–7 years, and finally “4” value given if the >7 years since previous birth.	PDHS
Diarrhea occurrence		1 = experienced diarrhea, 0 = otherwise.	PDHS
15.6-7.4,-1.8498ptDietary diversity		0 = < 5 food groups consumed, 1 = ≥5 food groups consumed (based on 8 food categories).	PDHS
Maternal characteristics			
Employment status		1 = employed, 0 = unemployed.	PDHS
BMI		“0” value given if mother is underweight (< 18.5 kg/m^2^), while “1” value given mother is normal or above.	PDHS
15.6-7.4,-14.8498ptEducation		“0” value given if there is no education, “1” value given if there is primary schooling, “2” value given if there is secondary education, “3” value given if there is higher education.	PDHS
Household characteristics			
Residence		1 = urban, 2 = rural.	PDHS
Region		“1” value given if province is Punjab, “2” value given if province is Sindh, “3” value given if province is Khyber Pakhtunkhwa, “4” value given if province is Balochistan, “5” value given if it is ICT, “6” value given if region is AJK, “7” value given if region is FATA.	PDHS
Wealth quintile		1 = poorest to 5 = richest.	PDHS
Drinking water source		1 = improved, 0 = unimproved.	PDHS
Sanitation type		1 = improved, 0 = unimproved.	PDHS
**PART B: Macro-level variables (World development indicators & UNDP, 2000–2022)**			
Dependent variable	Stunting prevalence	Percentage of children under five years with stunting.	WDI
National income		GDP is taken as per capita, which is PPP (in current US$)	WDI
Government health spending		Current spending on health expenditure (percentage of GDP)	WDI
Private health spending		Local or Domestic health expenditure as private (percentage of GDP)	WDI
Foreign health dependency		External per capita health expenditure (in current US$)	WDI
Poverty (Gini index)		Gini coefficient (current LCU)	WDI
Inflation		GDP deflator (annual %)	WDI
Human development Index (HDI)		Composite index (UNDP)	UNDP

Source: PDHS, Pakistan Demographic and Health Survey; WDI is World Development Indicators; UNDP is United Nations Development Programme.

In the PDHS 2017–18 dataset, 512 of 4,499 children under five were excluded due to missing anthropometric data required for the Composite Index of Anthropometric Failure (CIAF), leaving 3,987 children for analysis. To ensure full transparency of the research methods throughout the process, we produced a detailed sample screening flowchart, which recorded each exclusion step from the initial dataset to the final analytical sample; this chart is attached in **Appendix Figure 15**. The proportion of missing data for every piece of information collected in the study was very low. The lowest missing rate was for the children's sex, with only 0.5% missing; the highest was for mothers' BMI, with just 2.8% missing. Even for this item with the highest missing rate, fewer than three out of every one hundred study participants had missing entries, so the missingness of any single item alone could not be considered severe. However, if all study participants with any missing piece of information were excluded, and only samples with complete data were used to conduct the statistical analysis of the fully adjusted model, the sample size available for analysis would shrink substantially. From 4,499 initial children, 2,884 complete cases were retained after screening for SL-BN analysis. While excluding 832 cases with missing data left 2,052 considered for logistic regression. The results showed no statistically significant differences between the two groups — all chi-square test *p*-values for the comparisons exceeded 0.10, meaning that in terms of these core characteristics, there was no obvious bias between the samples that were retained and those that were screened out. Even after obtaining these non-significant results, we acknowledge that non-significant test results do not completely rule out the risk of sample selection bias. This is a recognized limitation of complete-case analysis, and we have elaborated on the limitations of this study in the Discussion section. The sample sizes for each stage of the analysis are detailed in **Table 6** (In **Appendix**).

### Outcome variable

The standardized indicators of child malnutrition were derived using anthropometric data on weight, height and age extracted from the 2017–18 Pakistan Demographic and Health Survey (PDHS). These indices are calculated in accordance with the WHO 2009 growth standards (29), where stunting is classified as height-for-age z-score (HAZ) < −2 standard deviations (SD) from the median. Underweight was also defined as WAZ under −2 SD, and wasting was defined as WHZ under −2 SD. These three anthropometric indicators (stunting, underweight and wasting) were combined to form the Composite Index of Anthropometric Failure [CIAF], where children were divided in seven different groups of malnutrition. The classifications range from no anthropometric failure to the presence of all 3 conditions (stunting, wasting, and underweight) simultaneously.

While the Composite Index of Anthropometric Failure (CIAF) can be analyzed as a categorical variable with seven distinct categories of malnutrition, we chose to dichotomize it into “any anthropometric failure” vs. ‘no anthropometric failure' for several reasons. First, the Bayesian network structure learning algorithm requires variables to be specified as either continuous or categorical; the seven-category CIAF would require a multinomial specification that substantially increases the parameter space and reduces statistical power. Second, the binary specification provides a parsimonious outcome that captures the overall burden of undernutrition, which is the primary policy-relevant measure for assessing the proportion of children requiring intervention. Third, this approach is consistent with previous studies examining child undernutrition in Pakistan (20, 21) and other low-income settings (22), facilitating comparability across studies. To ensure that important information is not lost, we conducted sensitivity analyses examining stunting, underweight, and wasting separately, which confirmed that the key determinants (wealth status, maternal education, environmental conditions) showed consistent associations across all three indicators (results available in **Appendix, Table 7**).

### Conceptual framework

The conceptual framework is that of UNICEF 1990 for nutrition of mothers and children (30), which has been analyzed in this study to assess various micro- and macro-level determinants affecting undernutrition among the child population. The framework is comprised of four interrelated levels:

(1) Enabling determinants, capturing macro-level governance, policy, political, social and financial actions;
(2) Underlying determinants related to household food security and individual socio economic factors such as access to clean water, sanitation and education;
(3) Immediate determinants focusing on dietary intake and health service availability; and finally,
(4) Outcomes reflecting physical growth/cognitive development outcomes that are influenced by these three preceding levels. Figure 1 depicts the operational framework utilized in this investigation.

**Figure 1:**
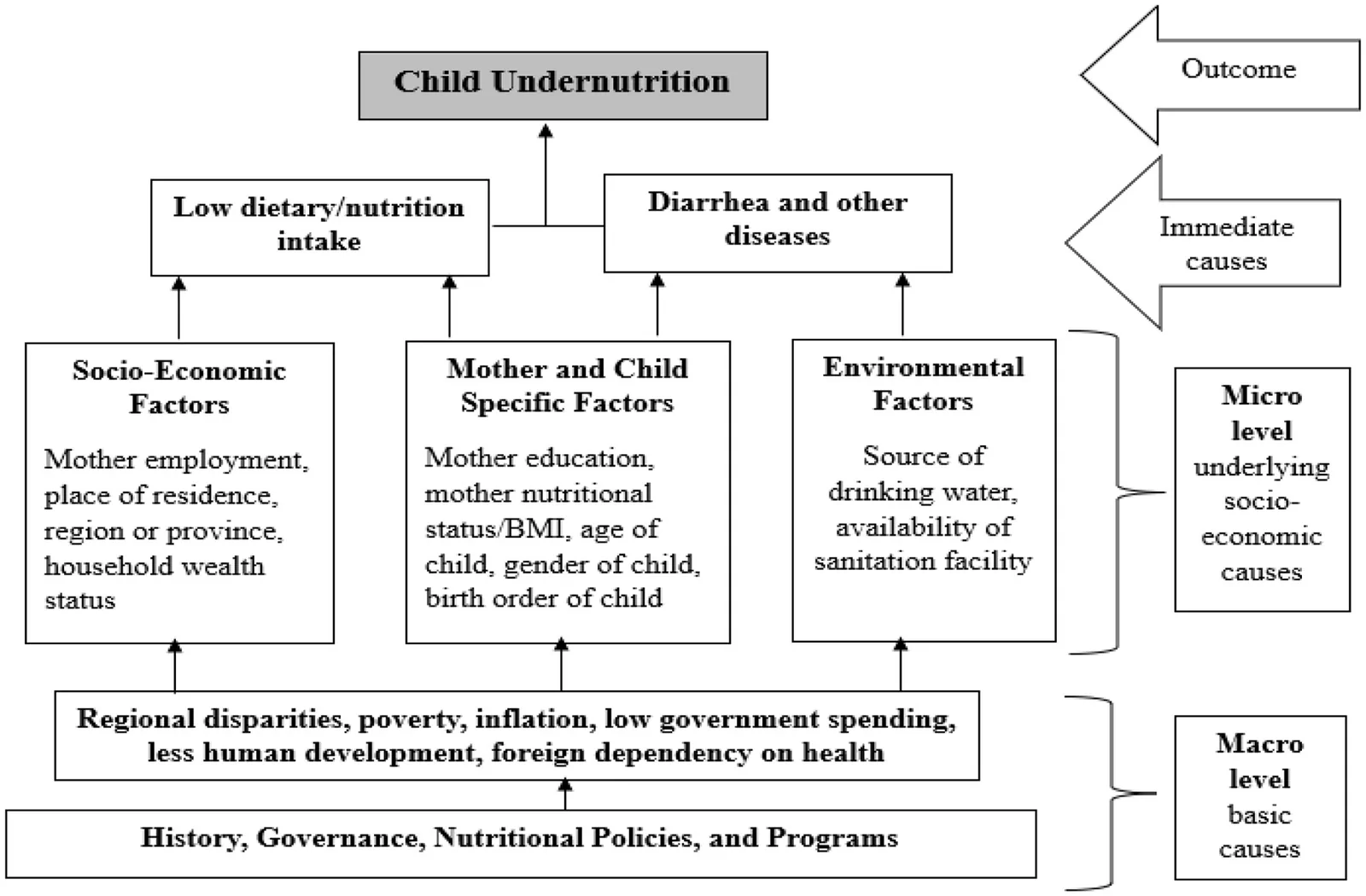
The theoretical basis for the factors that contribute to child undernourishment on both the macro and micro levels. Take note that the author adhered to the UNICEF Conceptual Framework on the factors that cause undernourishment in children and mothers (1990).

### Analytical approaches

#### Main model: structural learning-based Bayesian network (SL-BN)

Here, study treat child malnutrition as a contextual outcome and model the probability of such an event occurring using structural learning Bayesian Networks (SL-BN) that provide the likelihood model estimate of child malnutrition associated with a given set of inputs in this study. It brings together the intuition of classical econometrics with modern approach SL-BN. Traditional regression models, though interpretable, also impose strong assumptions of linearity, additivity and independence of predictor variables. Malnutrition is a multi-dimensional concept, but assumptions of these models are frequently falsified by consideration of malnutrition in all its forms. By contrast, traditional deep learning approaches (multi-layer perceptron; MLPs) are generally better suited to modeling complex non-linear interactions between many inputs and act primarily as predictive tools. But those models are typically black box and make them unable to actually shed light on what causes malnutrition conditionally or also reversibly.

In this work, we address this gap by proposing a Structural Bayesian Network (SL-BN) that enables one to learn a probabilistic graph model from the data. This strategy excels at representing non-linear and complex interactions between predictors, leading to interpretable Directed Acyclic Graphs (DAGs) with oriented edges among predictors that explicitly denote conditional dependencies; the set of variables under each edge of DAG is user-defined (31). A Bayesian network is a probabilistic graphical model that uses a Directed Acyclic Graph (DAG) to represent the conditional dependencies between individual random variables. Put simply, all edges in this graph are directed, and no closed cycles that connect back to their starting point exist. This graph can clearly sort out which random variables (the factors that require investigation and are subject to change) depend on which others, and under what conditions they are associated with one another. The core logic of this model is rooted in probability theory. When sorting out the complex associations between variables, it is supported by rigorous analytical methods, eliminating arbitrary speculative inference; when used for prediction, it can also convert the uncertainty in estimates into specific quantitative values, explicitly marking the “degree of uncertainty.” Structure-learned Bayesian networks (SL-BNs) inherently capture the stochastic dependencies between variables. They can not only calculate the estimated values of potential outcomes, but also attach standardized uncertainty metrics, which gives them unique applicability for assessing children's nutritional status and sorting out the various underlying influencing factors. This study specifically demonstrates how to build a model using SL-BN to simulate the probability of child malnutrition, while sorting out the probabilistic associations between various nutritional indicators and their core risk factors.

First, it is to clarify the terms throughout this paper. Structural Learning based Bayesian Network (SL-BN): A SL-BN is a graphical model that encodes conditional dependencies between random variables via a Directed Acyclic Graph (DAG) can be seen in Figure 2. This works fundamentally different from a weight-based Bayesian networks in prediction tasks. Our method is a type of structural Bayesian network in which the variables are represented by nodes and directed edges represent probabilistic dependencies. The graphical model corresponding to this structure is learnt from data using the PC algorithm, a method that detects and identifies conditional independence relationships between variables, yielding a well-known interpretable class of structures (DAGs) that can assist in an understanding of any underlying structural associations among determinants of child undernutrition. That distinction is important: Bayesian Networks are prediction models, but our Structural Learning based Bayesian Network aims to understand how various variables depend on each other and correlate with the outcome variable. These methods build a network, based on the directed edges between the nodes (edge direction) using either score — or any constraint-based approaches. The DAG represents the structural associations among random variables and is consistent with previous studies conducted in economic and finance domains where Bayesian networks have been applied to describe complex probabilistic dependencies and associations and decision-making systems (32–34). This structural modeling paradigm, as opposed to making estimates of the size of each association, is more appropriate if our research aim is to elucidate interactions between socio-economic, maternal and environmental determinants of child malnutrition.

**Figure 2:**
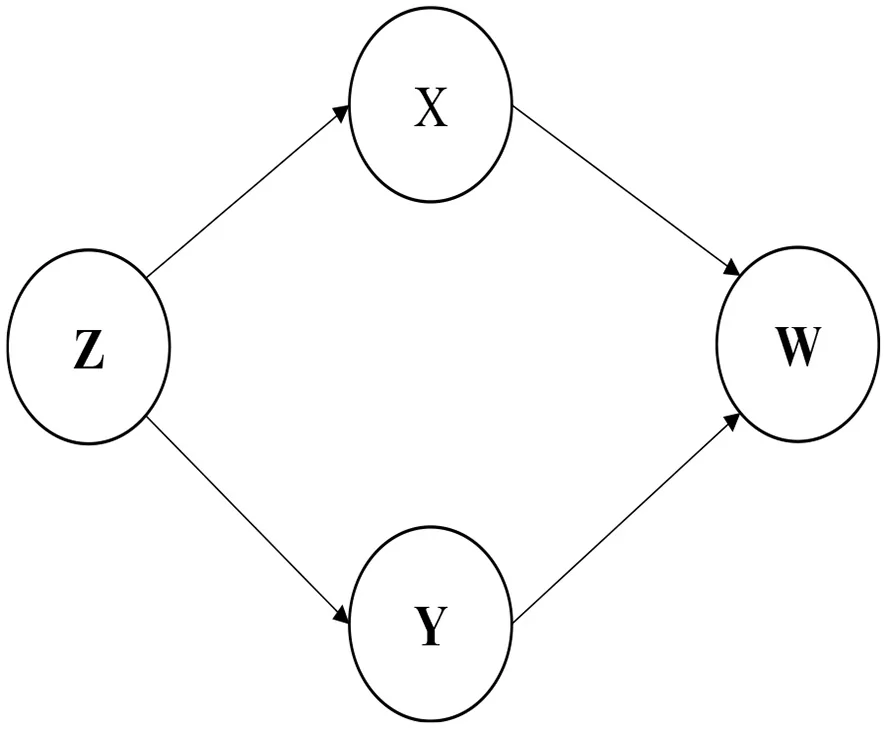
Directed acyclic graph (DAG).

One important point is, the research data this study use is divided into two categories. The first is the micro-level PDHS data, which is cross-sectional data. The second is macro-level data, which is observational time-series data. Both types of data have inherent limitations in their nature, which cannot support us in proving that there is a genuine causal relationship between variables. Secondly, the directed edges mentioned the SL-BN model we used, represent conditional dependence relationships at the probabilistic level and interpreted as structural relationships. This correlation does not conflict with potential causal paths, but it absolutely cannot be used to prove that these causal paths truly exist.

SL-BN is a very powerful tool for solving probabilistic problems with multiple mutually dependent variables. In this model, each variable corresponds to a node while directed edges indicate the causal or conditional dependencies between those nodes. Example: P(X,Y|Z) describes how the variable Z regulates the dependence between both X and Y (35).

##### Choice of structure learning and sensitivity analysis

Readers can appreciate the significance of pitted structure learning into their tasks by verifying our algorithm is relevant for them; therefore, a degree of sensitivity analysis was performed, especially comparing the construction SL-BNs from six different structure learning algorithms. These algorithms include the PC Algorithm, Chow & Liu Algorithm, Tan Random Function, Search and Score Algorithm, Clustering Functions and Hierarchical Functions.

Conducting full tests of robustness by testing subsampling methods of the bootstrap for each algorithm on network stability. As a result, we also rigorously quantified the accuracy of the output graphical models compared to true dependencies. More importantly, out of all the candidates the PC Algorithm was found to be the most efficient one with capability of returning models which fit closer to ground truth conditionally. Also, it had better graphical validation properties making it a superior candidate in this domain.

In contrast, the other five algorithms displayed major limitations. The trees constructed by the Chow & Liu Algorithm were very simplistic and did not account for more complex interdependencies in the data. On the other hand, while Tan's function allowed for tree-augmented dependencies it was still too limiting to be useful for this network being tested. On the technical side, Search & Score Algorithm was powerful in theory but too costly in practice and generally converged to local optimums resulting in rigidity of the networks. Since functions derived in this way from clustering do not distinguish between positive or negative functional correlations, they are limited in their utility for structural inference. Finally, the hierarchical functions produced highly complex structures that were either very difficult to interpret or did not generalize well across subsamples.

##### Estimation approach

After the algorithm had been chosen, estimation was performed with two different methods. The first approach is on a constraint-based framework where conditional independence is tested. This approach uses the chi-square test statistic in case of discrete variable or partial correlation for continuous variables, thus establishing edges present within the network. Then the PC algorithm identifies the network structure by connecting nodes iteratively on the basis of these tests in order to determine which node pairs are conditionally independent given other nodes.

The constraint-based PC algorithm, whose core function is to identify the structure of a network, relies on conditional independence tests to complete this task. The tests to be conducted are distinguished based on the types of variables: the chi-square test is used when processing discrete variables, and partial correlation analysis is used when processing continuous variables. After completing the aforementioned step, it is also necessary to determine the direction of the connected edges in the network, and collider discovery and Meek's rules will be used when determining the directions (36, 37). This method has been effectively adapted to a number of fields, such as genetics and finance, to learn complex dependency relationships (38, 39).

#### Detailed implementation settings for bayesian network analysis

All statistical and computational analyses involved in this study were completed using three professional tools. In Bayes Server 9.4, we used the built-in PC algorithm to complete the Bayesian network analysis for structure learning, strength of association and mesh query graphics; in addition, we exported three core statistical results from the network, which are mutual information, entropy, and directional dependence, respectively. Python 3.8 undertook another analysis task reusing the statsmodels and sklearn libraries within this programming tool to complete a multivariate logistic regression analysis with cross-validation. Finally, STATA MP 18.0 was used to complete three independent tasks: first, we generated descriptive statistical data with survey weights; next, we applied a logistic regression via the svy: logit command; and finally, we analyzed the numerical calculation of the Concentration Index.

##### Conditional independence tests

This study adopts the PC algorithm, with the parameter set for this algorithm as α = 0.05. When running this algorithm, two test methods are used, which respectively handle two completely distinct types of variables and are never mixed. Among them, the chi-square test is exclusively used to process categorical variables, while the partial correlation test is exclusively used to process continuous variables. The specific categorical variables include: Wealth Status, Region, Maternal Education, Residence, Water Source, Sanitation Type, Diarrhea Occurrence, Dietary Diversity, Child Sex, Child Age category, Birth intervals category. The specific continuous variables include: Mother BMI, Income, HDI, Government Health Spending, Private Health Spending, Foreign Dependency, Poverty, Inflation.

##### Discretization

For the micro-level categorical variables, no discretization was required as variables were already in categorical format (see Table 1). For the macro-level continuous variables, variables were analyzed using the Gaussian Bayesian network specification with the appropriate partial correlation test for conditional independence.

##### Missing data handling

Using listwise deletion for missing data yielded analytical samples of *n* = 2,884 for the Bayesian Network (14 variables) and *n* = 2,052 for logistic regression (19 variables). Comparisons of baseline characteristics (age, sex, region, wealth index) between included and excluded cases showed no systematic bias (*p* > 0.10). However, potential non-random missingness on unobserved characteristics remains a limitation. Macro-level data from WDI and UNDP had complete coverage for all variables in the 2000–2022 period.

##### Prior distributions

Non-informative Dirichlet priors were used for categorical variables (equivalent sample size = 1) and non-informative Gaussian priors for continuous variables (Jeffreys prior), following standard practice to allow the data to dominate the posterior distributions.

##### Model selection

The final network structure is selected against two criteria: the first is bootstrap edge stability, that is, the connections between all variables in the network, which have been tested through bootstrap analysis, are sufficiently stable; the second is the DAG interpretability under the UNICEF framework, that is, the directed acyclic graph corresponding to this network can clarify its underlying logic when placed within the framework of UNICEF. The study also adopted other algorithms to conduct sensitivity analysis, to verify whether the core associations identified by the PC algorithm are valid, and finally confirmed the robustness of these core associations. Furthermore, additional alternative guide for network structure taken by BIC which had the lowest Bayesian Information Criterion (BIC) scores (Macro: −2,314.8; Micro: −5,687.2), indicating the best balance of fit and parsimony.

##### Bootstrap stability

Edge stability was assessed using 500 bootstrap samples. Edges appearing in at least 60% of bootstrap samples were retained as stable relationships in the final networks.

##### Algorithm parameters

The PC algorithm was run with the following settings: maximum conditioning set size = 3 (to limit computational complexity while allowing for moderate-order conditional dependencies); significance level α = 0.05; and orientation rules based on collider discovery and Meek's rules for edge orientation.

The software, packages, and parameter settings used in this study are summarized in **Table 8 (Appendix)**.

### SL-BN model equational composition

In SL-BN we applied two approaches:

(1) Score-based through with we calculated strength of associations [Tables 2, 4];
(2) while Constraint-based method applied for graphical analytics [Figures 4, 9, and mesh query figures].

**Table 2:** SL-BN structure: strength of link between random variables in probabilistic Bayes network (Macro level analysis).

X	Y	Mutual information	Symmetric	Directional dependence: X | Y	Directional dependence: Y | X	Entropy X	Entropy Y
Poverty	Inflation	2.31625389	0.2628784	0.18430813	0.458215	12.56729	5.0549533
Human Development Index	Income	1.29258792	0.4079114	0.64779042	0.155117	−1.99538	8.3329715
Human Development Index	Private Spending on Health	1.18659823	1.9914953	0.59467287	0.372319	−1.99538	3.1870454
Poverty	Undernutrition	0.65384186	0.0803582	0.05202727	0.176433	12.56729	3.7058995
Inflation	Undernutrition	0.62341859	0.1423192	0.12332826	0.168223	5.05495	3.7058995
Income	Undernutrition	0.14160536	0.0235247	0.01699338	−0.038211	8.33297	3.7058995
Human Development Index	Undernutrition	0.13308958	0.1556130	0.06669887	−0.035913	−1.99538	3.7058995
Private Spending on Health	Undernutrition	0.12083026	0.0350591	0.03791294	−0.032605	3.18705	3.7058995
Foreign Dependency on Health	Undernutrition	0.00442527	0.0016855	0.00286426	−0.001194	1.54499	3.7058995
Government Spending on Health	Undernutrition	0.00031286	0.0001687	0.07476764	−0.000084	0.00418	3.7058995

Entropy values reported here are relative measures calculated within the Bayes Server framework and may take negative values because they are reported on a log-likelihood-based scale rather than as conventional Shannon entropy in bits. Mutual information (MI) quantifies the statistical dependence between two variables and is non-negative; therefore, MI indicates the strength of dependence but does not determine its direction. The columns “For X Given Y” and “For Y Given X” report directional dependence measures used to characterize the direction of the probabilistic relationship between the variables; positive values indicate a direct probabilistic association, whereas negative values indicate an inverse probabilistic association. These measures should not be interpreted as conventional conditional probabilities. The directional relationships represented in these measures are considered together with the orientation of the Directed Acyclic Graph (DAG) shown in Figure 4 to characterize the probabilistic structure of the network. Entropy and mutual information values were calculated using Bayes Server 9.4 and are reported using the software's log-likelihood-based formulation with natural logarithms rather than conventional Shannon entropy in bits. Accordingly, the reported entropy, mutual information, and directional dependence values should be interpreted as relative measures within the Bayes Server framework for comparing the strength and direction of probabilistic relationships among variables.

#### Score-based learning approach

A probabilistic-based Bayesian Network's goodness of fit can be assessed by finding the directed acyclic graph space with the best score. And stated that the posterior probability of the network Y structure, i.e., for dataset X is large along with the prior P(Y) (36, 37).


$$\begin{array}{l} P\left(Y|X\right)=\;\frac{P\left(Y,X\right)}{\sum_{Y^{\prime }}P\left(Y^{\prime },\;X\right)}=\frac{P\left(Y\right)\;P\left(X|Y\right)}{\sum_{Y^{\prime }}P\left(Y^{\prime }\right)\;P\left(X|Y^{\prime }\right)} \end{array}$$ (1)

Where: Y denotes the networking assembly (the Directed Acyclic Graph, or DAG) being evaluated; X denotes the observed dataset; P(Y|X) is the posterior likelihood of the network construction given the data; P(Y) is the previous likelihood of the network construction; P(X|Y) is the chance of the data given the network construction; Y' represents all likely alternative system structures in the search space; The denominator $\underset{Y^{\prime }}{\sum }P\left(Y^{\prime },\;X\right)\;$is the marginal likelihood, which normalizes the posterior probability over all possible network configurations.

Research asserts that identifying an optimal Bayes structure inside class N – P Hard is exceptionally challenging (40). Various methodologies, such as the K2 search algorithm and genetic algorithm, can be utilized to identify a suitable network. Several implemented scores can be utilized to estimate a SL-BN construction using the aforementioned equation.

#### Dirichlet Bayesian network scores

Supervised – in order to find the Y in the network from dataset named X while (θ vectors over important strictures), we need to maximize Dirichlet Bayes score. This optimization can be achieved using the following equation (41, 42).


$$\begin{array}{l} YX:\;{\;S}_{X}\left(\;Y\;\right)\;=\;\log\left(\;P\left(\;Y\;\right).\;\int^{p}\left(\;X|Y,\;\theta \;\right).\;P\left(\;\theta Y\;\right)\;d\theta \;\right) \end{array}$$ (2)

Given dimension for the area information/data were as:


$$\begin{array}{l} YX\;\left(Y,X\right)=\log\left(P\left(Y\right)\right)\;+\sum_{t=1}^{n}\sum_{k=1}^{n}\left(\log(\frac{G\left(T_{tk}\prime \right)}{G\left(T_{tk}+T_{tk}\prime \right)}\right)\\ \;+\sum_{n=1}^{r_{t}}\log\left(\frac{G\left(T_{tkn}+T_{tkn}\prime \right)}{G\left(T_{tkn}\prime \right)}\right)) \end{array}$$ (3)

Where *T*_*tkn*_ signifies the information numbers in the dataset in which the indicator *X*_*t*_ has its weightage and also parentages to *n*−*th* have value *k*−*th*. We assume that the vector (θ) probability distribution given the Y network is based on a Bayesian Dirichlet structure with hype parameterization $\alpha \;=\;\alpha_{tkn\;}\;=\;{T^{\prime }}_{tkn}$.

### Information concept notches

Study define BN as a method which conduit or channel featuring regulators that link vigorous or sedentary edges containing intricate knowledge (43). The information concept articulated through the subsequent scoring indices.

#### Entropy scores

Entropy measures the unavailability of information between random variables in structural learning-based BNs. This is done by specifically following a systematic, deterministic approach to minimize the entropy of random empirical data. The entropy metric (H(Y, X)), describing the Y network and the X dataset relationship, is formatted as follows (44):


$$\begin{array}{l} H\left(X,Y\right)\;=\;-T\overset{n}{\underset{t}{\sum }}\;\overset{q_{t}}{\underset{k}{\sum }}\;\overset{r_{t}}{\underset{n}{\sum }}\;\frac{T_{tkn}}{T}\log\frac{T_{tkn}}{T_{tk}} \end{array}$$ (4)

Whenever the parents of *X*_*t*_take the *k*−*th* value, the location where the *X*_*t*_ takes its *n*−*th* value begins to occur.

#### Mutual information scores

The mutual scoring method is practical in this study in direction to the observer the linking among *x*_*n*_ with *x*_*k*_ utilizing the command below (41).


$$\begin{array}{l} I\left(x_{n}{\;,\;x}_{k}\right)\;=\;\overset{i}{\underset{n,\;k}{\sum }}P\;\left({\;x}_{n}{\;,\;x}_{k}\right)\log\frac{\;P\;\left(\;x_{n},\;x_{k}\right)}{\;P\;\left(\;x_{n}\right)\;P\;\left(\;{\;x}_{k}\right)} \end{array}$$ (5)

The quantity of edges in a structural learning-based BN is denoted by i. The role of the PC algorithm is to learn the Markov equivalence class of graphs—that is, to identify a set of graphs that conform to specific common properties. To complete this learning process, it uses two types of tools. The first type is conditional independence tests, and the second type is the rules used to orient the edges in the graphs. This set of edge-orienting rules specifically includes collider detection and Meek's rules. This approach identifies a network structure that is consistent with the conditional independence relationships observed in the data.

### Constraint-based culture method

Constraint-based learning by PC algorithm is performed to find conditional dependencies among random variables. In studies, such a framework is adopted to appropriately design Bayesian networks using constraint-based Learning—these learn the conditional dependencies of random variables (45, 46).


$$\begin{array}{l} X^{\;2}\;=\;\overset{r_{Y}}{\underset{n}{\sum }}\overset{r_{X}}{\underset{k}{\sum }}\frac{{\left(P_{nk}-D_{n}^{k}\right)}^{2}}{D_{nk}} \end{array}$$ (6)

The predicted and desired frequencies are denoted by *P*_*nk*_ and $D_{n}^{k}$, respectively. The Partial Pearson Correlation (PPC) method allows for the successful application of the conditional independence test to continuous variables in direction to inspect the association amid these variables and random variables. According to a study the following formula might provide light on PPC statistical analysis (47).


$$\begin{array}{l} P_{pearson}\;=\;\frac{1}{2}\;\left|\;\log\frac{1\;+\;r_{\;X\;,\;Y|\;Z}\;}{1\;-\;r_{\;X\;,\;Y|\;Z\;}}\right|\;\sqrt{\;n\;-\;|\;Z\;|-\;3} \end{array}$$ (7)

In this context, n represents the size of the sample dataset and and *r*_*X, Y*| *Z*_ shows the PPC linked to the X and Y variables through conditioning Z. The number of variables used for conditioning in the X dataset is identified by Z.

### Policy and governance analysis: methodology

We employed a document analysis approach informed by the critical discourse analysis framework (Fairclough, 1995)^2^ to examine:

1. The historical evolution of nutrition policies and programs in Pakistan from 2000 to 2022;
2. The institutional frameworks and governance mechanisms for nutrition;
3. International donors and NGO roles in nutrition programming;
4. policy, political economy and nutritional status intersection.

#### Sources of document

All data included in this study must first meet the established Document Selection Criteria before entering the analysis stage. The specific selection criteria are as follows: The first category consists of nutrition policies released by the Ministry of Health and Planning Commission of Pakistan from 2000-2022; the second category is the Pakistan Demographic and Health Survey reports, with the corresponding years used being 2006-07, 2012-13, 2017-18; the third category includes nutrition reports released by the World Bank, UNDP, UNICEF, WFP, WHO, FAO; the fourth category comprises peer-reviewed articles focusing on Pakistan's nutrition policies; and the final category covers relevant news reports from Dawn, The News, The Express Tribune, Daily Times.

#### Analytical approach

There are also clear specifications for the thematic analysis process of the data: The analysis is undertaken by two independent researchers, both of whom use the same deductive coding framework constructed with reference to the UNICEF conceptual framework to conduct thematic analysis on all included data. The themes ultimately extracted from the data cover five areas in total, namely policy coherence, implementation difficulties, funding status, cross-sectoral collaboration, and equity. If the two researchers encounter differences in judgment during the coding process, they will reach a unified consensus through group discussion. A complete list of all data included in the analysis for this study is presented in **Table 9 (Appendix).**

### Robustness analysis: multivariable logistic regression with cross-validation

The hypothesis here is that childhood malnutrition affects a number of proximate indicators. To provide a robustness check of the results generated by our Bayesian network, we estimated a multivariable logistic regression model to assess the association between socio-economic indicators and child malnutrition status. This approach provides complementary estimates of the magnitude and direction of associations in the form of odds ratios, which are more familiar to policymakers and public health practitioners. Unlike the Bayesian network, which captures complex conditional dependencies, the logistic regression provides marginal effects that are easily interpretable and comparable to prior studies in the literature (48).

The binary logistic model is expressed as follows:


$$CIAF_{in}\;=\;Y_{in}\;=\;\{\begin{array}{c} 1\;If\;the\;child\;is\;malnourished\;\\ 0\;Otherwise\; \end{array}$$

In this binary state, *CIAF*_*in*_ and *Y*_*in*_ are equivalent. The response is recorded in the form of either a success or a failure, which signifies whether the malnourishment status of the young individual is confirmed (coded as 1) or not (coded as 0). In this study, Y denotes the child's nutritional state and its correlation with other explanatory variables (*X*_1_, *X*_2_, …, *X*_*n*_). Below is a reduced form multivariate logistic regression similar model specification for estimating odds ratios and probability scores for the outcome variable (CIAF) that is reliant on explanatory variables:


$$\begin{array}{l} P\left(Y_{i}=1\;|X_{1i},\;X_{2i},\;\ldots ,\;X_{kn}\right)=F\;(\beta_{0}+\;\beta_{1}X_{1i}+\beta_{2}X_{2i}\\ \;+\ldots +\beta_{n}X_{kn} \end{array}$$ (8)

The Yi, Xi, β's, and ε stand for the dependent variable CIAF, the explanatory factors, the coefficients that indicate the extent of link, and the error term, respectively, in this context. The results of the logistic regression are examined at three levels of significance: *p* < 1%, *p* < 5%, and *p* < 10%.

We adopted a commonly used method to test the model's performance, 10-fold cross-validation, and added a stratification process to this method. The basis for stratification is CIAF, the outcome indicator in the study. The core purpose of this approach is to ensure that after splitting the dataset, the proportion of children with malnutrition in each fold is completely consistent with the proportion in the original full study dataset, so that there will be no situation where a certain fold has an unusually large or unusually small number of children with malnutrition. We split observations using StratifiedKFold (shuffle = True, random_state = 42). Because this does not preserve PSU-level clustering, cross-validation metrics (Table 5) represent internal validation of prediction accuracy rather than a survey-design-consistent evaluation, a limitation exists here. Variance inflation factors (VIF) indicated no substantial multicollinearity among predictors in the logistic regression model (maximum VIF = 1.84 for wealth status; mean VIF = 1.36). The model performance metrics (accuracy, sensitivity, specificity, AUC) reported in Table 5 represent the mean values across the 10 cross-validation folds. We used the statsmodels tool in Python to estimate a set of logistic regression models, and adopted the maximum likelihood method for calculation. We additionally incorporated sampling weights and Huber-White robust standard errors to accommodate the complex survey design adopted in this study—this type of design is not the simple random sampling understood by the general public, and requires additional statistical processing to align with the actual circumstances of the survey. However, this set of analyses completed in Python has its limitations: it cannot fully address the issues of PSU clustering or stratification that may be encountered in complex surveys. To confirm that the core research conclusions remain reliable, we then conducted a robustness test using STATA's svy: logit command, remedied the previous shortcomings, and verified the stability of the conclusions.

### Additional test for inequality measurement – concentration index (CI)

In this study, we examine socio-economic disparities in child nutritional outcomes using wealth status at the micro level and the Concentration Index (CI) as a core analytical metric. Health disparity can be analyzed rigorously using either direct inequality techniques of order measurements or taking the ordinal data and converting it to a cardinal metric, and then applying standard inequality measures (49). Important health-related metrics like body mass index and anthropometric z-scores, including height-for-age (HAZ), weight-for-age (WAZ) and weight-for- height (WHZ), can serve as important measures to interrogate inequality. In addition, we can dichotomize health measures (e.g., malnourished vs. not malnourished) to address both the concentration of health and to compute a slope index of inequality (SII) on a latent scale ranging from [0, 1], representing health utility (50–52). This method combines the wealth-related health disparity measures using a ‘response category cut-point shift' approach (53, 54). In conclusion, through using the established health assessment framework (55), this study seeks to investigate wealth-related concentration indicators of child nutrition outcomes.

The Concentration Index (CI), a well-known health economics measure (56, 57), was used to assess wealth-related socioeconomic inequalities in nutritional outcomes for Pakistani children under five. The CI accurately measures inequality by showing the prevalence of dietary deficiencies across wealth groups.

CI was analyzed by following the below procedure and formula:


$$\begin{array}{l} C=\;\frac{2}{n.\;u}\left[\overset{n}{\underset{i=1}{\sum }}Y_{i}\;R_{i}\right]\;-1\; \end{array}$$ (9)

Every parameter in the formula has a clear definition: y_i_ is the measured nutritional outcome for each individual, μ is the weighted average of this nutritional outcome, R_i_ is the wealth distribution quantile rank assigned to each individual after the aforementioned sorting, and n is the total sample size of the current study.

If the nutritional outcome to be analyzed is a binary variable with only two possible values [1, −1], after the initial CI is calculated, the value must also be adjusted using the Wagstaff correction method. The correction formula is $c_{corrected}=\frac{c}{1-u}$, which adapts to the value constraints of binary variables and avoids deviations that occur when an uncorrected Concentration Index is applied to non-continuous variables. Subsequent calculations of the standard error and 95% confidence interval are completed in the STATA software. The calculation process uses the delta method and survey-based robust variance estimation to match the complex sampling design of this study. All these calculations incorporate the PDHS sampling weights from start to finish, with the specific calculation method for the weights being v005/1,000,000, to ensure that the results can represent the overall population level.

In addition to the Concentration Index, we also calculated the Slope Index of Inequality (SII). This index is used to measure the absolute difference in malnutrition status between the population group of the poorest and the group of the wealthiest, and it is also calculated using a specified regression formula: *y*_*i*_ = *a*+β*R*_*i*_+ϵ_*i*_. The β coefficient obtained from this calculation is the value of the SII.

Using individual-level data, this study applied the Concentration Index (CI) to assess wealth-based important inequalities in child nutritional status. Taken with the y-axis, it shows cumulative percentage of child undernutrition and on the x-axis is a curve showing wealth-deciles of the population ranked by wealth. The Concentration Index (Ci) is equal to double area among the equality line (which is 45° degree), and concentration curves, which has a theoretical boundary of −1 to +1 (56, 58). A CI of 0 means no SES-related disparity. Where basic relative measures reflect inequality between two populations, the Concentration Index (CI) summarizes inequality between more than two population groups and does so by analyzing the distribution of an outcome variable—child undernutrition in our case—along wealth gradations. Wealth status, the ranking variable, is either continuous or ordinal: from poorest to richest. A CI above zero means that undernutrition is more frequent among the wealthier groups, whereas a negative CI reflects higher rates of occurring in poorer groups.

## Results and discussion

### PART A-Macro-Level: Macro-analytical perspective on the underlying causes of child undernutrition

#### Policy and governance failures and successes in addressing undernutrition: a critical macro-level discussion

For a long time, the Government of Pakistan has failed to come up with a set of specific and feasible measures to address the malnutrition problem of its people. At present, most of the nutrition-related projects within the country are short-term projects funded by the UN and INGOs. The existence of these projects exactly exposes two core problems: first, the government has not taken the leading power of nutrition work into its own hands from a strategic level, nor has it taken the initiative to lead and be responsible for this work from the level of national strategy; second, there is simply no unified and coherent national nutrition work framework across the country, and a complete, mutually coordinated national nutrition work system has never been established from start to till date (59). This adds, in the context of post-devolution, an important dimension of exploring nutrition-related resource constraints and limits within the system while working to ensure vertical integration for both historical and contemporary nutrition initiatives (including funding), multi-sectoral engagement as well as monitoring and evaluation systems. Within provincial administrations, poor horizontal coordination between departments and weak connections between programs, beneficiaries and sectors are major hindrances. While the Punjab Health Department mostly concentrates on monitoring and treatment initiatives, other departments have failed to perform their duties adequately. Such a situation calls for setting up nutrition departments at the district level to improve governance and optimize nutrition programs (27).

The history of national nutrition policies of Pakistan have not ensured consistency or require specific research and development. This is evident through the introduction of several initiatives like card-based rationing along with food distribution schemes, and subsidies for lower-income households by targeted wheat distributions especially among flood-affected populations in Punjab. Furthermore, nationally managed cash transfer programs such as the Benazir Income Support Program (BISP) have sought to assist underprivileged women (60). UN agencies like FAO, UNICEF, WHO, and WFP have led nutrition-related work with provincial health ministries. These initiatives have created Baby-Friendly Hospitals and Nutrition Corners in hospitals to provide dietary help (27). As part of the Safe Motherhood Program, government health agencies give pregnant women edible oil during and after pregnancy. After the 2010 floods, the Management for Acute Undernutrition at Community-level (CMAM) effort was created to combat undernutrition with severe and moderate levels (SAM).

In flood-prone regions of South Punjab, nutrition is frequently subordinated to the health bureaucracy due to insufficient financing, exacerbating the workload of Lady Health Workers (LHW) and impeding the screening for severe acute malnutrition (SAM) due to deficiencies in field personnel training (61). Restricted social capital, corruption in therapeutic meal distribution, extensive travel distances, logistical challenges, and mother stigmatization further impede therapeutic access. The World Health Organization sanctioned the Expanded Programme on Immunization (EPI) alongside the Lady Health Workers Programme, while the Maternal, Newborn, and Child Health (MNCH) Programme persisted. In 2010, the World Food Programme and UNICEF initiated a biennial vitamin A supplementation initiative via the Expanded Programme on Immunization to avert childhood blindness. Moreover, Nutrition interventions including Deworming by LHWs of MI- INGOs were also implemented during mother and child health weeks in different districts across Punjab, along with fortification of wheat flour through UK Aid.

There is evidence that the manufacturers of formula milk engage in unacceptable marketing and distribution of their product, which exacerbates child undernutrition by preventing breastfeeding (62, 63). Research by Save the Children (2013) found that 84% of mothers reported being advised by healthcare professionals to use formula milk: a powerful campaign promoted worldwide through these marketing practices (64). The Punjab Food Authority (PFA), in response, has introduced the 16 regulations to prohibit nefarious marketing tactics for formula milk, with input from the Formula Milk Association but approved by the Punjab Assembly. Some of these new regulations include banning the distribution and sale of infant formula in hospitals, as well as sugary drinks from being sold on school property. Through about 700 field teams and 2,000 food technologists, the PFA monitors compliance with ingredient approval, scientific panel assessment, and the labeling of all imported formula products in Urdu (65, 66).

Government efforts to bring inter-departmental and multisectoral approaches to tackling hunger are often limited by low institutional capacity, bureaucratic inefficiency, and endemic corruption. Many departments of the government continue to be dys-functional and under-performing, making it extremely difficult for any pro-posed policy to be implemented effectively (27). These structural weaknesses are exacerbated by a political apparatus that maintains systemic violence and naturalizes social exclusion against the most marginalized sectors. According to Escobar (1995), hunger was not just about food availability, but rather fundamentally a policy problem that could only be addressed with sustainable policies to deliver food security for disadvantaged sectors (67). In Pakistan for instance, the destitute often suffer due to their distance from power structures and social networks. These inequalities are further amplified by extreme disparities in income and levels of poverty that are greater than comparable between South Asian countries like India, Bangladesh, and Sri Lanka (68). Moreover, customary income support programs by the World Bank have been shown to be somewhat disconnected with Pakistani social and cultural realities. Women in a purposive measure to the movements of social capital lost husbands their wives most likely, that together with people with disabilities and extreme poverty-stricken were for any number entrenched structural and cultural barriers in the national socioeconomic structure which existed will hesitate much less against. As a result of this, these social safety nets are mostly available to the wealthy while low-income people are systematically left behind.

In Pakistani financial assistance programs (69), for example, such selection criteria often exclude widows and female household workers. Identifying priorities for development and poverty reduction may reinforce rather than disrupt existing power hierarchies (70–72). However, even in models that control for socioeconomic disadvantage, BISP selection is strongly associated with political party and local patronage network affiliation (69). The humanitarian assistance system is highly competitive for limited resources and therefore, systematically excludes so many people though they may be equally vulnerable (73). Participants in South Asia have past lived experiences as part of households and communities on the wrong side of historical structural and social systems that discriminated against them in obtaining government support, so it is vital that processes to distribute funds are carefully designed to identify those who qualify through criteria related to social exclusion (72). One may also note that even perceptive analysis of Pakistani bureaucracy states, through extensive dysfunction and politicization from within the system, “move a stuck-up file” while those who do not exist within the system have to slog hard merely to “put a wheel” on it (74). These dynamic cements the fact that the bureaucratic function of political and economic power, degenerates into a process for funding, which is automatically skewed coming from these same mechanisms put in place.

Supporters of the Benazir Income Support Programme (BISP) argued that with the BISP, some of Pakistan's poorest women would no longer receive 5,000 rupees a month to over five million beneficiaries who will be taken out of poverty. Nonetheless, continuing ineffectiveness of the administration in executing the programme has rattled this lofty intention. But the scheme has faced controversy and allegations of corruption, including claims of vote-buying in political interference over which organizations get to help. The NADRA audit of the beneficiaries' database was carried out in 19 December 2019, followed by board approval from BISP. That audit uncovered hugely flawed management of the programmes day to day running. Using data analytics, the exercise found 142,556 beneficiaries were government employees (2,543 of them in grades 17–21). It also noted that a significant number of recipients traveled abroad — 153,302 had taken at least one international trip and 10,476 traveled multiple times. 691 BISP beneficiaries and 43,746 of their spouses have one or more vehicles registered in their names (75, 76). The results are a disturbing trend of systematic corruption and rundown businesses funded by an annual budget allocation of Rs. 100 billion that should have used to help the poorest members of our society but instead allocated to political patronage and personal gain.

CMAM, a community-based approach to the management of acute malnutrition is an essential response to severe malnutrition but ultimately socio-economic empowerment and education of women offer a sustainable long-term solution for maternal and child under nutrition (77, 78). Research suggests that multisectoral approaches, despite their perceived merit, fail in implementation especially in post-devolution era with lack of implementation and interdepartmental co-ordination. As such, targeted, micro-level interventions — nutrition-specific and nutrition-sensitive — are important elements of well-designed social safety nets for low-income women (15). But the geography and socio-economic terrain is largely uncharted. Policies evolve over time as the relative strengths of lobbies shift, and government decisions and spending will be invariably biased in favor of those with greater resource — both money and political voice. Consequently, there is a huge disconnect between the lofty aspirations presented in policy papers and their effective implementation on the ground. Not only is this oversight exacerbating regional inequities, it also highlights structural failures that make it nearly impossible for many in low-income communities to have equal access to food and health services.

Meanwhile, India and almost all of its neighbors, as well as vast swathes of sub-Saharan Africa are mired in permanent nutritional deprivation. One of the most blatant examples is Pakistan, where colonial history and ongoing social turmoil continue to mold the current state of nutrition in this region. Colonial and post-colonial authorities dispossessed communities of natural resources, displaced ecosystem-specific knowledge systems and culinary traditions rooted in ancient cultures, established structural inequities that persist in the present (4). On the one hand, such policies created a stalemate, on the other, fostered dependency on foreign aid while eroding societal resilience. As a result, local communities have been administered continuous neglect and want through these socio-political spaces. Recent scholarship highlights the tendency of dominant neoliberal and public health discourses to misidentify malnutrition as purely a character flaw rooted in cultural practice, and fails to recognize where deeper historical and political currents have enabled its continuation (27). Such a remedy for hunger cannot be made without an urgent, realignment of historical colonial relationships at the root of both and can neither be spoken nor enacted without recognizing the social/economic inequalities their inextricable connection creates between us (now, starting now).

Previous strategies also have the obvious shortcoming of widely missing a clear application to the complexities of Pakistani social and cultural-political realities, which has created inequitable distribution of essential nutritional resources and services. “In practice, such programs tend to operate in an aura of detachment from the lived experience of the populations that they are supposed to serve — experiences that most often don't address both the pernicious force of political power and blatant power over wealth here.” Those who have intervened in these localities have grossly failed to acknowledge the impact of colonial legacies, neocolonial dependencies, neoliberal policy architectures and entrenched regional inequalities on structural vulnerability. Critical nutrition-relevant domains —including but not limited to water justice, food systems, land reforms, gender equity and reproductive health, public-health access, and therapeutic treatment programs and social safety nets—have largely skirted the deep-seated disparities of class/caste/power hierarchies. Consequently, policies and initiatives have largely overlooked the influence of social and cultural capital, structural violence, and historical marginalization on consolidating nutritional inequalities (27). Nutrition governance must be reconceptualized in a new way: using a framework that questions the structural drivers of malnutrition.

Chronic poverty and increasing inflation have severely limited food choices and dietary diversity among low-income populations in Pakistan. Though global interventions in the form of an introduction of CMAM (Community-Based Management of Acute Malnutrition) and a distribution of RUTFs (Ready-to-Use Therapeutic Foods) have been advocated and presented as a rapid technical solution, it has failed to be resilient with socio-cultural aspects. Such approaches are often located within broader neoliberal governance agendas that emphasize an increasingly minimal redistribution of social and economic inequalities (79). At provincial level, the Multi-Sectoral Nutrition Coordination (MSNC) mechanism (as per well intentioned plans and designs) also failed to serve as an all-encompassing solution. Rather, it exemplifies larger trends in which narratives of development from the Global North are used to justify interventions elsewhere in the Global South. Study found the unintended consequences of external development efforts: increased bureaucratic disorder, poverty and institutional inefficiency; producing a higher demand for development strategies that are reductive and remove them from the historical, political & cultural conditions of local communities (80).

One of the most important public health issues in Pakistan is malnutrition, which has a disproportionately high impact on women and children residing in remote geographic and underprivileged socioeconomic areas. This segment comprises people living in water-stressed areas, menial workers with fixed wages, household workers, low caste communities as well as unemployed youth and illiterate women that have high fertility rates along with heavy physical tasks. These gross disparities highlight the need for policy interventions to deal with caste- and class-based stratification at the district level as well as for dealing with rural-urban divides within provinces, alongside ethnic-regional inequities (27, 81). The worst part is, almost 50 per cent of people in some rural districts (these are the most remote and unfurnished) are still far away from the Lady Health Worker (LHW) program. This very large coverage gap highlights persistent challenges in management capacity and systemic weaknesses contributing to inequalities in the access of life-saving health and nutrition services (82).

At macro-level the drivers of malnourishment in Pakistan require political, legal and institutional reforms guided through culturally relevant context-specific solutions as urged for by the World Bank. Good story, but from the standpoint of providing an analytical framework a different story is revealed; neo liberalization and privatizing activity configures cheaper commodity access to basic needs—food, water and health care—for poorer households. Consequently, services intended to provide equitable access tend to remain chronically under resourced and regulated. On the other hand, health and nutrition services remain largely unaffordable even for clients targeted by the private sector (27). If scientific/market-driven solutions linked to larger global objectives have remained in fashion, policies struggled to engage in the ground realities of Pakistan's entrenched though anemic socioeconomic structure. Thus, rather than solving the problems or bending social hierarchies for a change, such solutions perpetuate exclusion and catalyze the call to explore structural determinants of under-nutrition among children. All statements in this analysis referring to corruption, political patronage, and governance failure are sourced from formally recorded media reports, government audit reports (e.g., NADRA's and BISP audit report), and academic literature which are publicly available. The collected contents only serve as qualitative background information. The purpose is to explain contextual information rather than being derived through numerical calculation; its sole purpose is to help readers smoothly understand the conclusions reached by this study's quantitative analysis. These contents are in no way the conclusions empirically derived via this study's core research method, Bayesian network analysis. The statements related to corruption, political patronage, and governance failure have never been independent research outcomes derived and calculated from actual data through this set of analytical methods. The key themes of the findings presented in this section are also illustrated in Figure 3.

**Figure 3:**
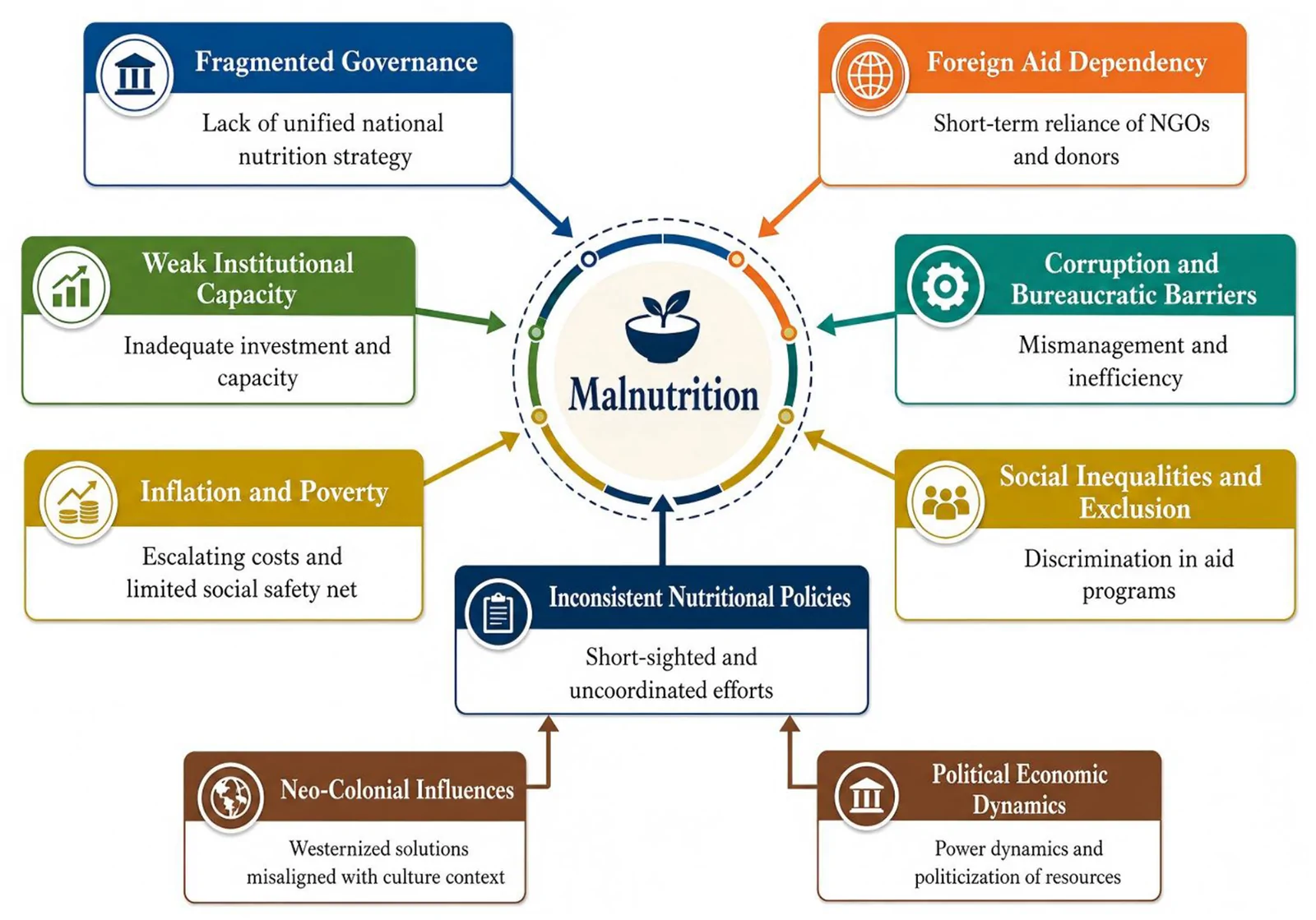
Some Key factors contributing to malnutrition based on rigorous investigation of failure in policies and governance in combating hunger and malnutrition.

#### Macro-level SL-BN model justification and stability assessment

The macro-level sample size used in this study is very small, totaling only 23, spanning 2000–2022. When conducting data analysis, a small sample size can easily lead to a problem called “overfitting”—that is, the model only fits patterns to the tiny set of samples it is given, and becomes completely inaccurate when faced with a new set of data. Beyond that, we were also concerned that the analytical model we built was not reliable enough, producing unstable results that fluctuated sharply. To avoid these problems, and to genuinely test the model's stability, we implemented several preventive measures.

First, we strictly limited the variables used in the macro network model to seven pre-specified predictors and one outcome indicator (stunting prevalence or child malnutrition). These indicators were not chosen arbitrarily; they were finalized step by step with reference to the framework of UNICEF and relevant academic literature. This avoids adding extra burden to the already small sample size, and firmly defines the scope of the content to be analyzed from the outset. Second, we ran 500 bootstrap iterations, and only variable associations that appeared in at least 60% of these iterations were retained for the final analysis. The actual stability percentages for key edges ranged from 51.8% (Foreign Dependency → Undernutrition) to 96.2% (Poverty → Undernutrition); full details are reported in **Table 10 (Appendix)**. A special note must be made here: the data used in this study are time-series data arranged in chronological order, and the successive samples in such data are inherently correlated. However, the bootstrap method inherently requires all observation samples used for calculation to be mutually independent, so the underlying assumptions do not align. Therefore, the results obtained through the bootstrap method can only provide exploratory descriptive reference, and cannot be used as formal statistical evidence to prove the model's robustness. Third, we conducted a sensitivity analysis using several other different network estimation algorithms—in other words, we recalculated the data using several different analytical methods to check whether the results would change. We ultimately confirmed that the main conclusions drawn using the core PC algorithm did not change significantly when the analytical method was replaced, and the results produced by all algorithms were consistent and stable.

Finally, we must clarify two key limitations to avoid misunderstanding: First, all associations observed in this study are only exploratory probabilistic associations, meaning these variables are highly likely to co-occur, but they are not definitive causal conclusions. It cannot be said that a change in one variable will definitely lead to a change in another. Second, limited by the short time-series span of this study's dataset, we were unable to carry out rigorous time-series modeling, and could not sort out the logical sequence of variables' influences along the timeline. This also means that future research will need to use data with a longer time span or higher collection frequency to verify whether these associations are truly valid.

#### Structural learning-based bayesian network results macro-level drivers of child undernutrition

Structural learning-based Bayesian Networks (SL-BN) operates on two broad paradigms in order to learn optimal network architectures: by constraint and score approaches. With these methods, the probabilistic Bayesian network can analyse conditional dependencies and describe those joint probability distributions of our random variables in a Bayesian manner. Therefore, this allows a more natural depiction of the multidimensional dependencies within the data. Moreover, derived from a limited time-series dataset (*n* = 23 annual observations, 2000–2022), these macro-level findings are strictly exploratory. Due to small sample size and temporal dependence, they represent probabilistic associations rather than causal effects, and must be interpreted with this limitation.

Figure 4 depicts the network structure of SL-BN for macro-level variables, demonstrating the complex relationships among key sociodemographic predictors of child undernutrition in Pakistan. The Representation of the SL-BN shows that child nutritional outcomes are conditional for predictors income and private health spending, also as is indicated with percentage independence impact of Human Development Index. Moreover, the model demonstrates that child undernutrition is additionally conditionally affected by inflation: a finding corroborated by the percentage effect of poverty rates. This network diagram explains the complex conditional relations inherent to child nutrition as it relates to socio-relative situations.

**Figure 4:**
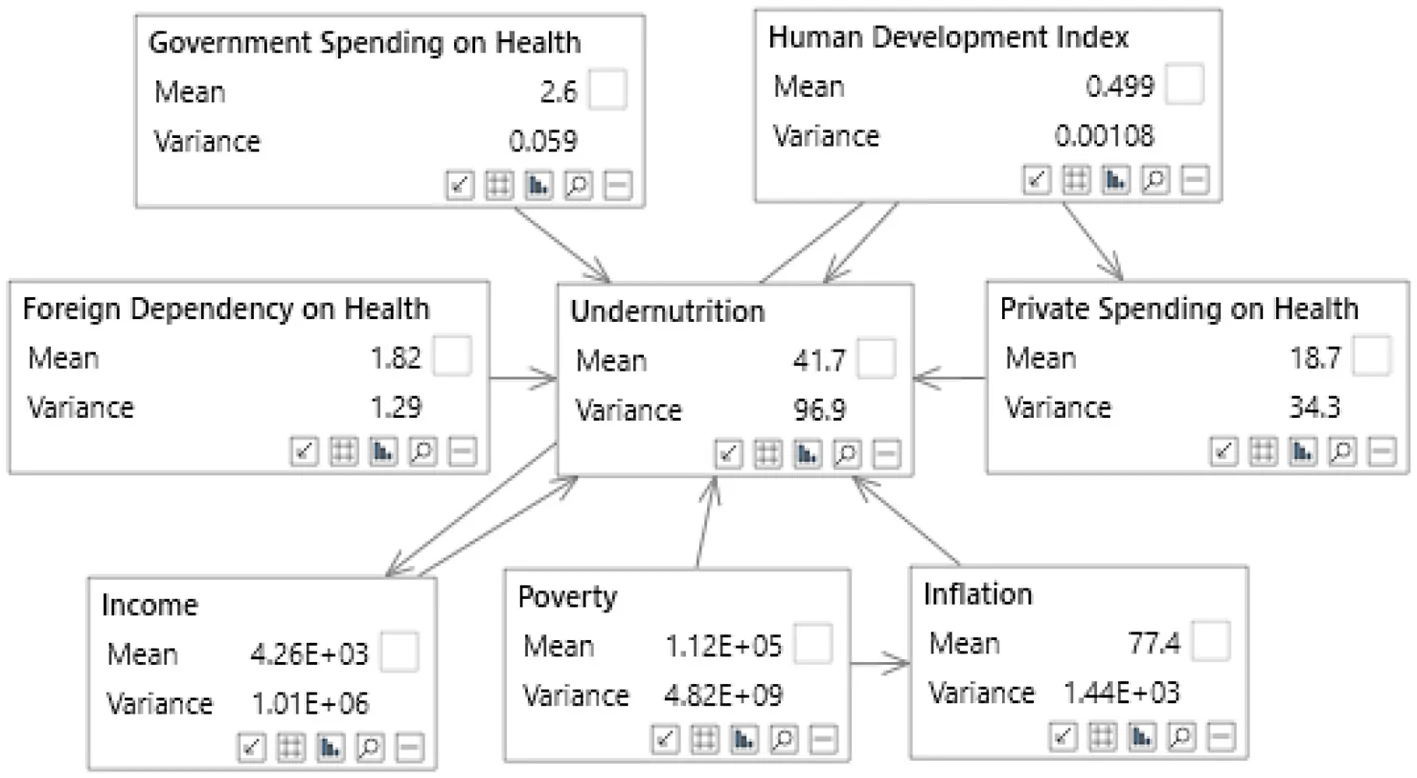
An architecture of SL-BN from a probabilistic structural learning approach (macro-level).

**Figure 5:**
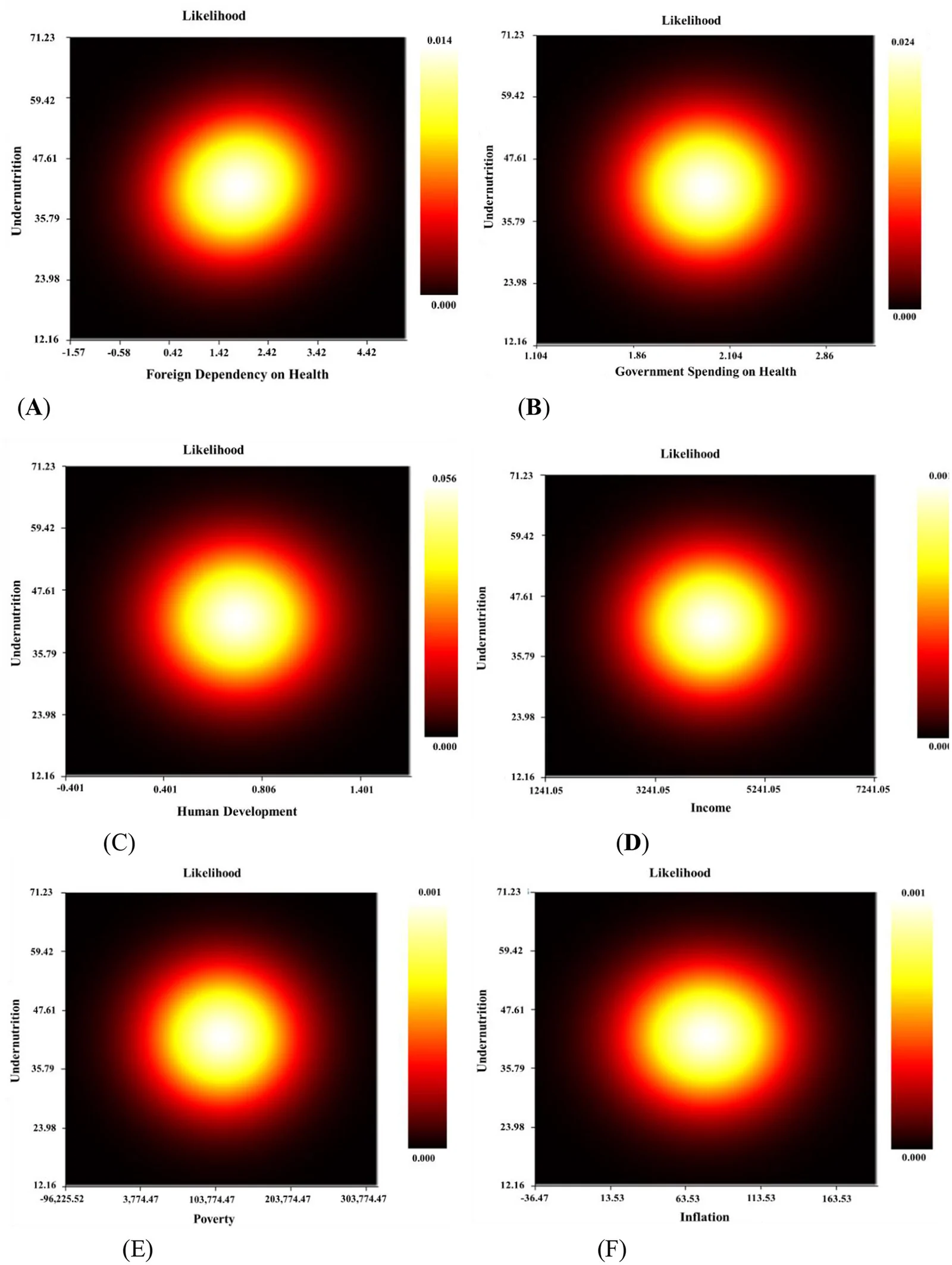
Relationship between macro level determinants and child undernutrition, designated by mesh query. **(A)** Health dependency on foreign support. **(B)** Health investment by government. **(C)** Human development. **(D)** Income status. **(E)** Status of poverty. **(F)** Status of Inflation. Mesh Query heatmaps visualize the joint probability distribution of two indicators. Yellow and red indicate high and low probability densities, respectively. Ellipses represent confidence contours (68%, 95%, and 99%). Ellipse tilt denotes relationship direction (upward = positive; downward = negative), while flatness indicates association strength (flatter = stronger; circular = independent).

Utilizing mutual information and symmetric score, as well as inter-node entropy metrics (NMI) and conditional probabilities (“Y given X” and “X given Y”) to examine macro-level factors that affect children undernutrition (or child stunting) (Table 2), it depicts SL-BN architecture. The symmetry scores and mutual information quantify how strong the relationship between two variables is, while the conditional probabilities explain how directional these interactions are. The flow of information between nodes is further assessed by entropy scores. First, we need to explain a statistical indicator used here, called the mutual information score. Its function is to measure how close the probability-based association between two sets of paired variables is. The problem of child malnutrition within Pakistan only exhibits a weak negative correlation with residents' income. Simply put, overall, the higher residents' income is, the lower the local proportion of child malnutrition will be, but the strength of this inverse association is very low. A rise in income will not bring about a substantial reduction in malnutrition; the statistical value used to quantify this correlation is MI = 0.1416, and directional dependence = −0.0382. It is not only income that shares a similarly weak negative correlation with malnutrition; the negative correlation between malnutrition and the Human Development Index is also weak. The Human Development Index is an indicator that measures the overall development level of a region, and a higher index represents better local development. Even if the development level improves, the local proportion of child malnutrition will only decline by a very small margin, and the corresponding statistical value is MI = 0.1331, directional dependence = −0.0359. The study adopted the SL-BN analysis method, and also measured several other groups of similarly weak negative correlations: these are private medical expenditure, corresponding to MI = 0.1208; government medical expenditure, corresponding to MI = 0.0003; and the degree of dependence of medical expenditure on foreign capital, corresponding to MI = 0.0044. All these factors follow the same pattern: the more money individuals spend on medical care, the more funds the government invests in medical care, and the less a country's medical expenditure relies on foreign capital, the lower the proportion of child malnutrition will be. However, the strength of all these correlations is very low, and improvements in these factors cannot bring about a large-scale improvement in malnutrition. In contrast to these weak correlations, the positive statistical associations between malnutrition and the two factors of inflation and poverty are very strong. That is, the more severe inflation is and the larger the impoverished population is, the higher the local proportion of child malnutrition will be. Moreover, the magnitude of this impact is considerable, rather than being an insignificant weak association. The MI value associated with inflation is 0.6234, and the MI value associated with poverty is 0.6538.

All these research findings point to the same conclusion: higher income, better development, and greater medical input have only very weak correlations with a reduction in child malnutrition. However, economic hardship is positively associated with of children facing undernutrition. This economic deprivation limits people's ability to access sufficient food, medical services, and essential medicines, directly placing children at risk of undernutrition.

The variable nodes within the network topology enlightened several conditional dependencies among macro-level variables: (1) the child undernutrition in particular was connected to income and private health spending within the learned network structure, while inflation was also linked to child undernutrition, with poverty contributing to the observed directional dependence. The SL-BN topology further revealed conditional dependencies among the macro-level variables. In particular, child undernutrition These network relations specify that the probability distribution of child undernutrition is associated with blends of socioeconomic and macroeconomic conditions rather than with lonely predictors. these directed network relationships should be interpreted as probabilistic dependencies represented by the learned DAG rather than as causal effects.

##### Joint probability distributions (Macro level)

The Figure 3 show the macro-level visualization graphs generated by this mesh query perform a simple function: it collates various macro conditions that can influence outcomes with the prevalence of child undernutrition, calculate the probability of their co-occurrence, and map these probability distributions onto the graphs one by one, allowing the correlation between the two to be understood at a glance. Each small cell in every graph is marked with a color transition ranging from yellow to red. The depth of the color directly represents the frequency of the co-occurrence of the two factors in that group—the more concentrated the red and yellow patches in a region, the more frequently the group of factors appear together, and the higher the probability of their linked occurrence. An ellipse representing the probability distribution is drawn in each cell. If the ellipse is tilted, its direction of tilt reveals the direction of the correlation between the two factors; the flatness of the ellipse, that is, its eccentricity, can directly quantify the strength of the correlation between the two—the flatter the ellipse, the stronger the correlation, and the closer it is to a perfect circle, the weaker the correlation.

First, examine Group A. This graph specifically plots the joint distribution of dependence on foreign medical aid and child undernutrition. The probability distribution in the graph is highly dispersed, with no trend of clustering whatsoever, featuring an extremely low concentration level (MI = 0.0044), indicating that the correlation between the two is negligible. However, the ellipse tilts upward, meaning the two factors are positively correlated—the more a region relies on foreign funding to support its medical expenditures, the higher the local prevalence of child undernutrition tends to be. This result runs completely counter to ordinary intuition. The emergence of this counterintuitive outcome may stem from the fact that foreign aid itself is mostly directed to regions that already face more prominent nutrition problems and have more urgent needs. Next is Group B, which examines the relationship between the government's own medical expenditures and child undernutrition. The shape of the ellipse in this graph is close to a perfect circle (MI = 0.0003), indicating that the two factors have almost no correlation. The variable of government medical expenditure plays a very limited role when used alone to explain why children experience undernutrition. Following that are Groups C and D. These two graphs present the joint distribution of HDI and child undernutrition, and income level and child undernutrition, respectively. The ellipses for both groups tilt downward, indicating a negative correlation in both cases: regions with higher HDI and higher local overall income face a lower risk of child undernutrition. The eccentricities of the ellipses for both groups are at a moderate level (with corresponding MI values of 0.1331 and 0.1416, respectively). This result supports a clear conclusion: human capital development and local economic resources have a negative probabilistic association with children's nutritional status. Unlike the trends of the previous groups, Groups E and F display the joint distribution of poverty level and child undernutrition, and inflation level and child undernutrition, respectively. The ellipses for both groups tilt upward, indicating a positive correlation in both cases: regions with higher poverty rates and higher local inflation levels face a higher risk of child undernutrition. Moreover, the eccentricities of the ellipses for these two groups are greater than those of all previous groups (with corresponding MI values of 0.6538 and 0.6234, respectively), further confirming that economic deprivation and unstable prices are positively associated with children's nutritional status.

All the patterns summarized from these visualization graphs are fully consistent with the mutual information scores and conditional dependence relationships in Table 2. Taken together, all this content completely presents the joint probability structure between various macroeconomic variables and child nutrition outcomes within Pakistan.

#### Discussion on macro-level drivers of child undernutrition

This analysis provides a broad national context for understanding the macro-level drivers shaping child under-nutrition in Pakistan. This article integrates a cross-country analysis of nutrition policies from a number of key publications between 2000 and 2023 with data from the World Development Indicators (WDI) and United Nations Development Programme (UNDP), which spans 2000–2022. It will provide a longer time series and a more robust but even playing field for the next generations of epidemiologists in terms of morbidity and mortality—through the MDGs and SDGs, two large international development frameworks which set global goals to follow socioeconomic/health progress (83). This, however, becomes quite evident when one looks at the nutrition challenges of Pakistan in light of broader sustainable development efforts.

SL-BN modeling uncovers two main inverse associations: per capita income with child under-nutrition, and the Human Development Index (HDI) with child under-regulation. Results show that improved development prospects and human capital creation are largely responsible for mitigating malnutrition risk. This finding is congruent with previous studies reporting GDP per capita and HDI has negative probabilistic association with under-five mortality, thereby improving general population health (84–87). Additionally, we find that increasing domestic health expenditures whether public or private has a large negative association with childhood malnutrition further adding to the impetus for tackling this problem. This discovery supports the current literature establishing a positive association between healthcare spending and health outcomes, resulting in lower morbidity among children (88–91).

The analysis also, for the first time, shows that development assistance negatively correlates/associated with lower probability with rates of child under nutritional states in developing countries points as evidence that this form of international aid can improve health systems and related services in said nations. Yet, a recent study from Pakistan shows how culturally inappropriate interventions imposed externally may limit the capacity of local institutions to develop. Moreover, economic stressors—earlier in its cycle such as inflation and rise of poverty—are correlated with higher levels of childhood malnutrition. These economic pressures fuel a vicious cycle of declining health status and reduced demand for, as well as access to food with high nutritional value (92). Such results corroborate findings from earlier studies that have shown an association between economic shocks, price changes and unhealthy dietary behavioral outcomes as maternal child mortality (93–96).

The research shows that addressing child malnutrition crisis has to identify root causes which are mostly the socio-economic disparities and omnipresent structural fragility deeply entrenched in Pakistani society. This points the way toward urgent investments in targeted human capital development and continuing support, both of which can produce substantial and lasting improvements in child health outcomes.

### PART B-Micro-Level: Exploring micro-level drivers and disparities in child undernutrition

#### Descriptive statistics

A principal instrument to identify occurrence and prevalence of malnutrition throughout populations globally standardized. Part 1 of this analysis forms the first part of a two-piece investigation, with ways in which z-score distributions and malnutrition rates create some of the key context for it to follow. Then we can compare it with published reference values, commonly analyzed in scientific studies and gain a better insight into what undernutrition really is. More specifically, this approach highlights the differences between demographic groups and what may explain this heterogeneity. As seen in Figure 6, there was a wide range of z-scores showing the differences that will allow us to understand nutritional deficits and its root causes for this population.

**Figure 6:**
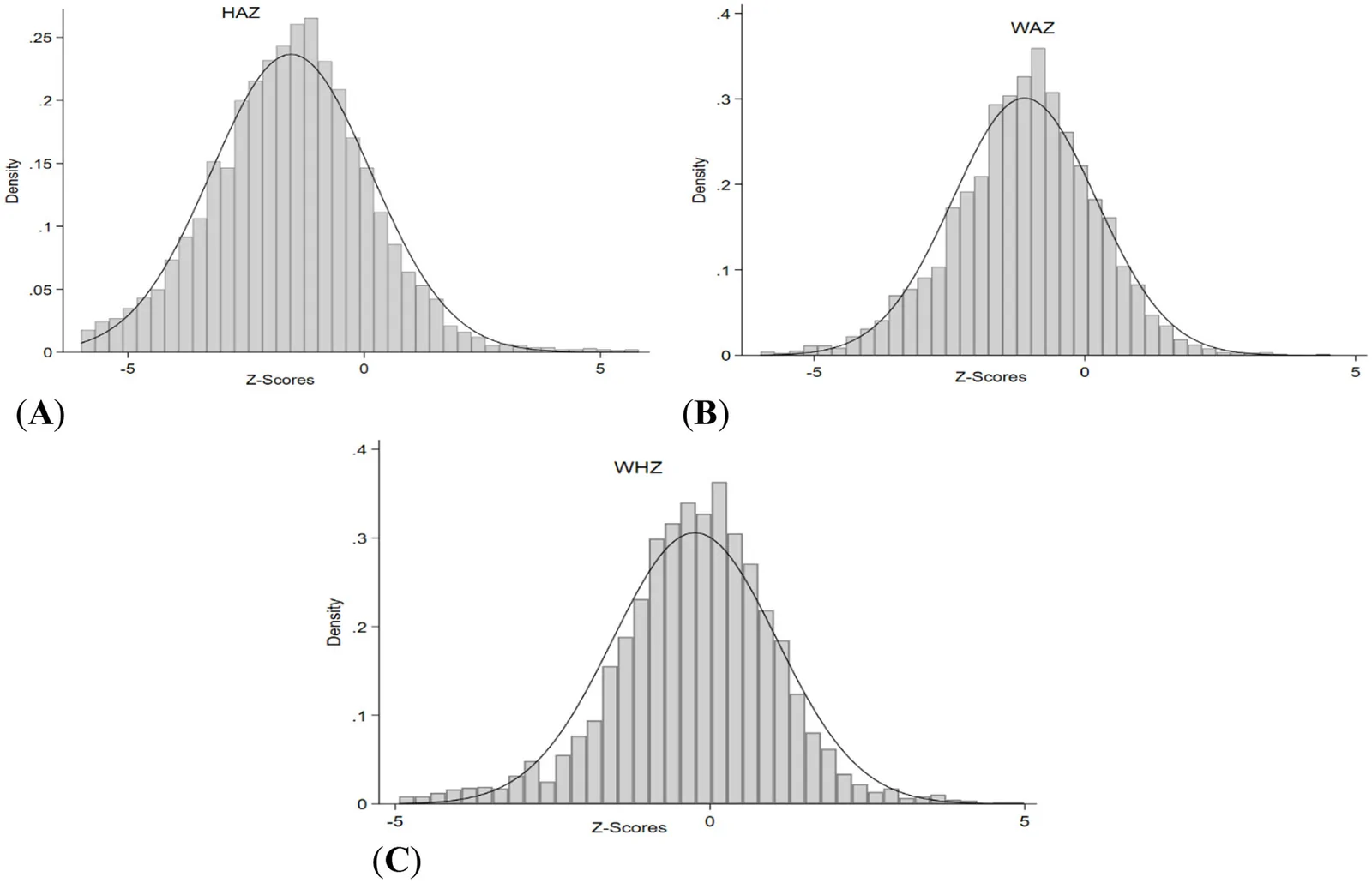
Distribution of z-scores. **(A)** The assessment of HAZ (i.e., Stunting). **(B)** The assessment of WAZ (i.e., Underweight). **(C)** The assessment of WHZ (i.e., Wasting).

The prevalence of acute malnutrition by WHZ is low (Figure 7). In comparison, the prevalence of stunting and underweight is significantly high. When weight-for-age and height-for-age are analyzed with respect to weight-for-height, a positive correlation is observed, further indicating interlinked growth dimensions. Yet, the Pakistan Demographic and Health Survey (PDHS) findings reveal a weak association of weight-for-age with height-for-age. The difference indicates that these two anthropometric measures are determined by different underlying factors in the population.

**Figure 7:**
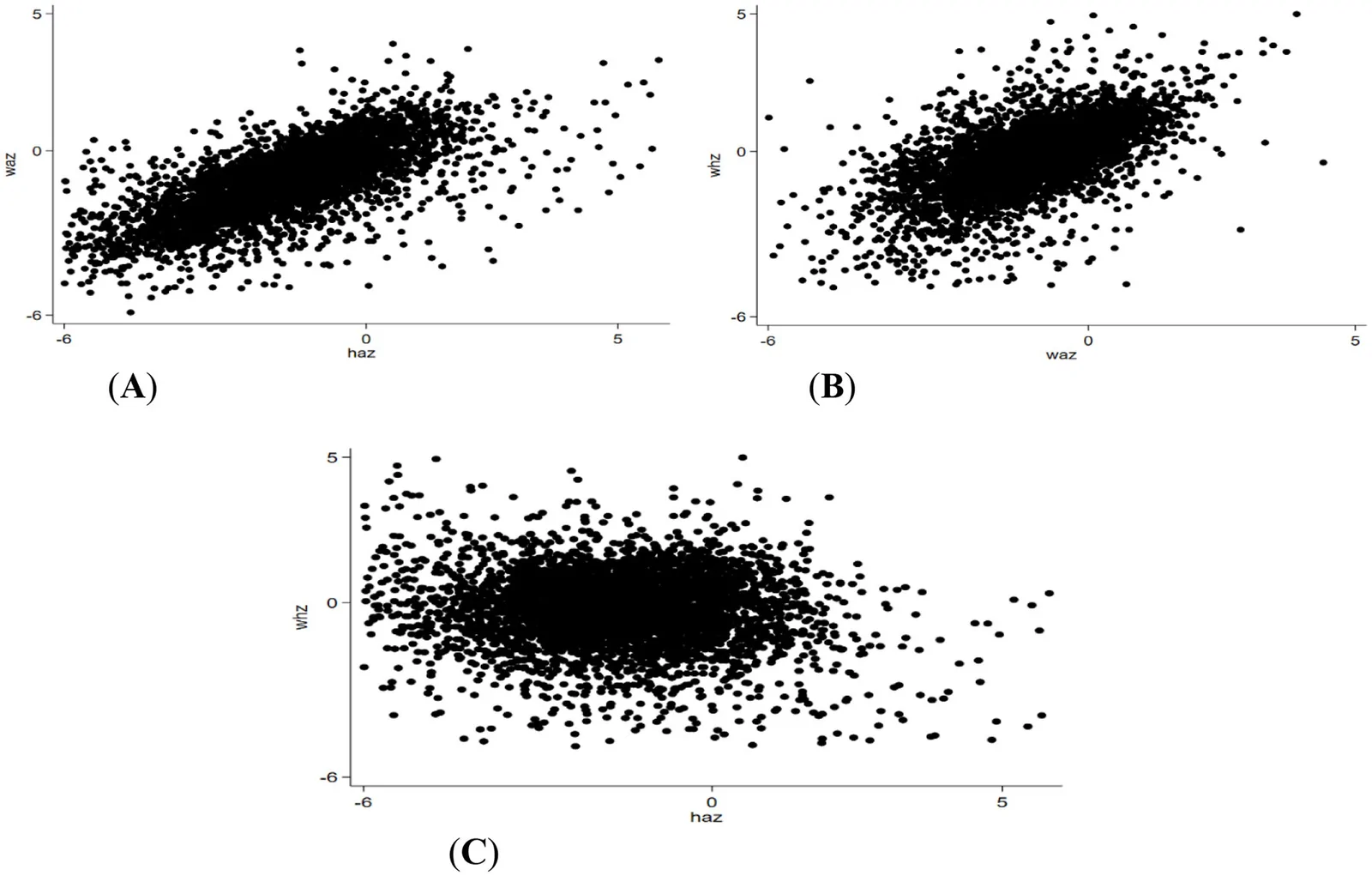
Different anthropometric indicators showing correlation. **(A)** Link among WAZ & HAZ. **(B)** Link among WHZ & WAZ. **(C)** Link among WHZ & HAZ.

To better understand malnutrition, it is necessary to examine mean z-scores within narrow age bands defined by month. This method consisted of recoding the continuous age variable into categorical ranges and plotting mean z-scores by anthropometric indicators. In Pakistani children, the z-scores for height-for-age and weight-for-age (derived from DHS data), decline sharply starting at around 20 months of age (Figure 8). On the other hand, weight-for-height z-scores display a more stable association throughout this age range and best capture patterns of growth faltering and acute malnutrition.

**Figure 8:**
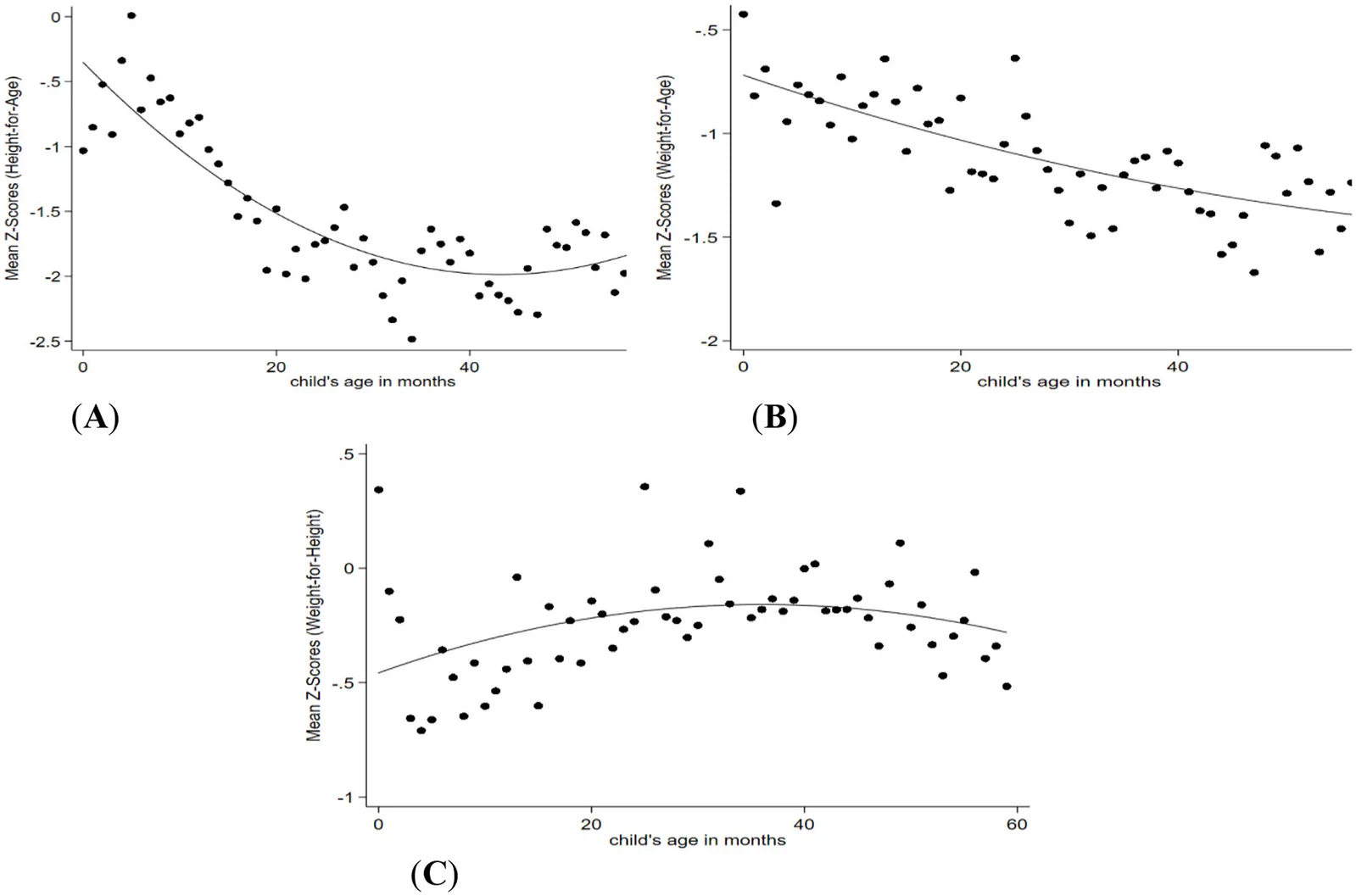
Mean/average z-scores of the various anthropometric predictors across ages in months. **(A)** Mean z-scores height-for-age. **(B)** Mean z-scores weight-for-age. **(C)** Mean z-scores weight-for-height.

Table 3 details child undernutrition (CIAF) prevalence by socio-demographic category, showing its systematic concentration among disadvantaged groups. Malnutrition rises with age, from 24.8% (< 12 months) to approximately 48% (25–48 months) (*p* < 0.001). Regionally, prevalence is highest in Balochistan (51.2%), Sindh (49.8%), and FATA (48.5%), and lowest in Islamabad (21.5%) (*p* < 0.001). Prevalence decreases from 52.5% in the poorest wealth quintile to 20.5% in the richest, and from 45.2% among mothers with no formal education to 21.5% for those with higher education (*p* < 0.001). Rates are also higher in households with unimproved water (44.2% vs. 28.5%) and sanitation (46.5% vs. 26.8%). Lastly, maternal underweight (BMI < 18.5 kg/m^2^), recent diarrhea, and low dietary diversity (< 5 food groups) significantly elevate malnutrition prevalence (*p* < 0.001).

**Table 3:** Showing survey-weighted bivariate relationship (prevalence) among socio-economic indicator with childhood undernutrition status.

Characteristic	Category	Weighted *N*	Prevalence of CIAF (%)	95% CI	χ^2^ *p*-value
Child's sex	Female	887	38.2	(34.1–42.3)	0.68
	Male	921	39.5	(35.4–43.6)	
Child's age (months)	< 12	284	24.8	(20.2–29.4)	< 0.001
	13–24	325	44.1	(39.2–49.0)	
	25–36	423	48.3	(43.5–53.1)	
	37–48	427	47.6	(42.8–52.4)	
	49–60	349	45.2	(40.2–50.2)	
Birth intervals	≤ 2 years	767	36.2	(32.0–40.4)	< 0.001
	3–4 years	547	40.5	(36.2–44.8)	
	5–7 years	377	44.8	(40.0–49.6)	
	>7 years	117	47.5	(42.0–53.0)	
Recent diarrhea	No	372	28.5	(24.0–33.0)	< 0.01
	Yes	1,436	45.8	(42.5–49.1)	
Residence	Urban	734	28.5	(24.8–32.2)	< 0.001
	Rural	1,074	43.8	(40.5–47.1)	
Province/region	Balochistan	305	51.2	(45.5–56.9)	< 0.001
	FATA	189	48.5	(42.0–55.0)	
	Sindh	416	49.8	(44.5–55.1)	
	ICT	63	21.5	(15.0–28.0)	
	Khyber Pakhtunkhwa	288	39.2	(34.0–44.4)	
	Punjab	297	34.8	(29.5–40.1)	
	Gilgit-Baltistan	108	28.5	(22.5–34.5)	
	Azad Jammu & Kashmir	142	30.2	(24.5–35.9)	
Drinking water source	Unimproved	1,430	44.2	(41.0–47.4)	< 0.001
	Improved	378	28.5	(24.0–33.0)	
Sanitation facility	Unimproved	1,284	46.5	(43.2–49.8)	< 0.001
	Improved	524	26.8	(22.5–31.1)	
Maternal BMI	Underweight (< 18.5)	181	50.2	(44.0–56.4)	< 0.001
	Normal or above	1,615	31.5	(28.5–34.5)	
Maternal education	No formal	1,146	45.2	(42.0–48.4)	< 0.001
	Primary	235	36.5	(31.0–42.0)	
	Secondary	288	30.8	(26.0–35.6)	
	Higher/graduate	139	21.5	(16.0–27.0)	
Maternal employment	Not employed	1,579	42.5	(39.5–45.5)	< 0.001
	Employed	229	30.2	(25.0–35.4)	
Household wealth	Poorest	550	52.5	(48.0–57.0)	< 0.001
	Poorer	507	42.8	(38.5–47.1)	
	Middle	324	36.2	(31.5–40.9)	
	Richer	254	28.5	(24.0–33.0)	
	Richest	173	20.5	(16.0–25.0)	
Dietary diversity	< 5 food groups	1,425	48.5	(45.5–51.5)	< 0.001
	≥5 food groups	383	28.5	(24.5–32.5)	

The table represents the survey-weighted prevalence of CIAF within each category of exposure along with 95% CI adjusted for survey weights. For example, 48.3% of children aged 25–36 months were malnourished (CIAF = 1). Percentages represent subgroup prevalence, not the total malnourished proportion. Statistical significance is based on design-adjusted Wald chi-square tests: ^***^p < 0.001, ^**^p < 0.01, ^*^p < 0.05.

#### Output of structural Bayesian network for micro-level causes of child undernutrition

At micro-level, the analytical framework utilizes a complex Bayesian network structure that feeds dual-learning approaches into both stable (mathematical) and visual representations for data processing. The element of score-based learning provides the measuring gauge for strength of relationships between stochastic variables in a network topology by returning measurable estimates of their probabilistic dependencies. This analytical approach enables accurate estimation of association among variables, while ensuring that the architectural structure of the network is maintained via constraint-based parameters. In order to provide a clearer view of the probabilistic relationships of the conditional dependencies among these variables, we adopt a well-founded constraint-based learning strategy which not only focuses on the directional (either positively correlated or negatively correlated/associated with lower probability) dependence but also both structural independence and conditional independence that are found in a Bayesian network. By employing all these techniques together, both the clarity of how variables interact with each other and the robustness of how those interactions are formed become much greater.

We also show the network architecture of the structural learning-based SL-BN used for micro-level data in Figure 9. This number highlights the immense relationship recognized between socio-economic factors and child undernutrition at the country level in Pakistan. The SL-BN found that children's nutritional outcomes are conditionally reliant on the socioeconomic status of their household. This is evidenced by the percentage impact of the mother's employment position. Moreover, it has been established that maternal education is a conditional factor influencing the child's nutritional status. This is evidenced by the proportional impact of household affluence and maternal employment. Moreover, the model indicates that children's nutrition is conditionally dependent on the mother's body mass index (BMI), which is affected by the household's financial resources. Environmental factors, especially water and sanitation conditions, exert a conditional influence on a child's nutritional quality, as demonstrated by the percentage impact of home wealth. Water and sanitation conditions are of paramount importance. This approach elucidates the myriad and interconnected elements influencing child undernutrition at the micro level.

**Figure 9:**
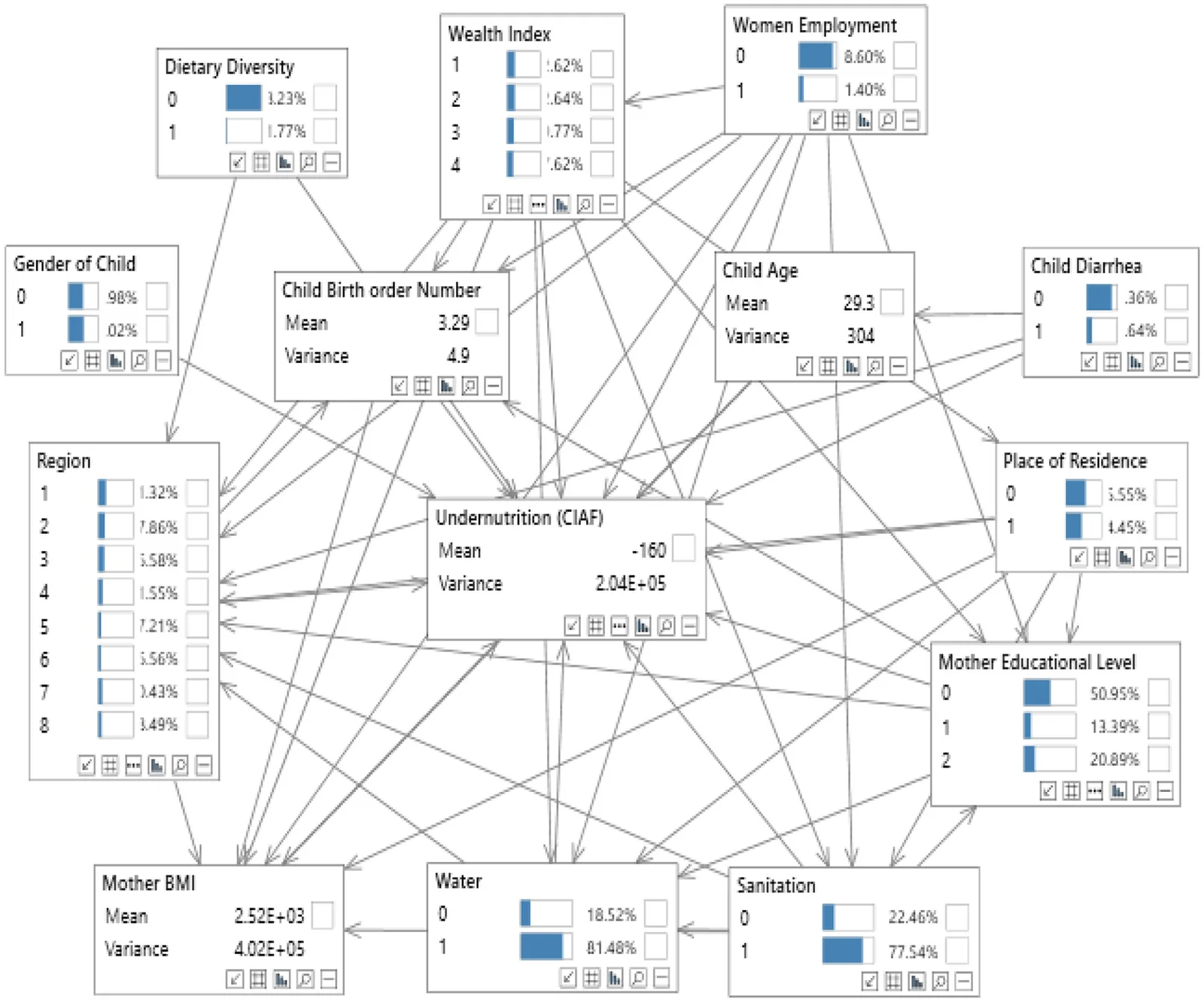
Probabilistic Bayes intelligent network structure (Micro-level analysis).

Table 4 presents the entropy metrics, conditional probabilities (“X given Y” and “Y given X”), symmetric scores, mutual information, and the implementation of an advanced structural Bayesian network (SL-BN) about micro-level drivers of child undernutrition. These metrics quantify the rate at which information is transferred across the network and between its component parts, especially when used with variables specific to each neuron in a grouped manner: entropy scores provide an insight into how fast information transfer occurs at all levels of the hierarchy among the graphs; mutual information and symmetric scores interrogate variable relationships (or joint distributions) among sets of neurons. The “Y given X” scores expose a strong negative probability relationship between malnutrition in children (measured by the Composite Index of Anthropometric Failure — CIAF) and dietary diversity. This indicates that increased dietary diversity among community children significantly reduces the odds of malnutrition in Pakistan. Likewise, the rise in household asset ownership and maternal employment are inversely associated with malnutrition, which may represent increased economic capital for securing a balanced diet.

**Table 4:** Random variable association strength within the context of a Bayesian network (micro-level).

X factor	Y factor	Mutual scores information	Symmetric outcome	Directional dependence: X | Y	Directional dependence: Y | X	Outcome of Entropy for X	Outcome of Entropy for Y
Wealth status	Maternal Educational Level	0.2154028	0.15248657	0.134391659	0.176212356	1.6027989	1.22240465
Wealth index	Place of Residence	0.1356103	0.11844907	0.084608433	0.19740446	1.6027989	0.68696676
Wealth index	Sanitation	0.10618646	0.09944964	0.066250646	0.199342589	1.6027989	0.53268327
Mother educational level	Birth intervals	0.10534619	0.06131735	0.086179471	0.047588447	1.22240465	2.21369247
Wealth index	Region	0.09987823	0.05547707	0.062314889	0.049991492	1.6027989	1.99790467
Region	Undernutrition (CIAF)	0.09354617	0.01963345	0.046822139	0.01242089	1.99790467	7.53135789
Wealth index	Mother BMI	0.08753394	0.0184795	0.054613176	0.011121313	1.6027989	7.87082745
Mother educational level	Region	0.07994968	0.04965341	0.06540361	0.040016763	1.22240465	1.99790467
Wealth index	Birth intervals	0.06645766	0.03482658	0.041463508	0.030021182	1.6027989	2.21369247
Region	Mother BMI	0.05862071	0.01188009	0.029341096	0.007447846	1.99790467	7.87082745
Water	Undernutrition (CIAF)	0.04974687	0.01242045	0.103829557	−0.006605299	0.47912053	7.53135789
Place of residence	Mother Educational Level	0.04268184	0.04470774	0.062130865	0.034916293	0.68696676	1.22240465
Sanitation	Mother Educational Level	0.04081669	0.04651242	0.07662469	0.03339049	0.53268327	1.22240465
Child diarrhea	Undernutrition (CIAF)	0.03560399	0.00890408	0.076424059	0.004727433	0.4658741	7.53135789
Wealth index	Undernutrition (CIAF)	0.03326396	0.00728342	0.020753672	−0.004416728	1.6027989	7.53135789
Sanitation	Region	0.03274111	0.02587629	0.061464503	0.016387725	0.53268327	1.99790467
Place of residence	Sanitation	0.03115102	0.0510819	0.045345747	0.058479443	0.68696676	0.53268327
Place of residence	Region	0.02927726	0.02180906	0.042618167	0.014653984	0.68696676	1.99790467
Region	Birth intervals	0.02283647	0.01084456	0.011430208	0.010316006	1.99790467	2.21369247
Sanitation	Water	0.02121329	0.04193162	0.039823454	0.044275472	0.53268327	0.47912053
Place of residence	Mother BMI	0.01713794	0.00400522	0.024947256	0.0021774	0.68696676	7.87082745
Water	Region	0.01633025	0.01318538	0.034083813	0.008173691	0.47912053	1.99790467
Sanitation	Mother BMI	0.01624135	0.00386537	0.030489699	0.002063487	0.53268327	7.87082745
Women employment	Region	0.01453748	0.01235773	0.040965556	0.007276365	0.35487088	1.99790467
Wealth index	Water	0.01138495	0.01093698	0.007103171	0.023762192	1.6027989	0.47912053
Sanitation	Undernutrition (CIAF)	0.00892166	0.0022127	0.016748529	−0.001184602	0.53268327	7.53135789
Mother educational level	Undernutrition (CIAF)	0.00725698	0.00165803	0.005936645	−0.000963569	1.22240465	7.53135789
Child diarrhea	Child Age	0.00725264	0.00305849	0.015567821	0.001695827	0.4658741	4.27675865
Women employment	Mother Educational Level	0.00432006	0.00547788	0.012173609	0.003534067	0.35487088	1.22240465
Dietary diversity	Region	0.0042063	0.00403121	0.047281091	0.002105357	0.08896375	1.99790467
Birth intervals	Mother BMI	0.00341092	0.00067647	0.00154083	0.000433363	2.21369247	7.87082745
Women employment	Wealth Index	0.0031995	0.00326868	0.009015943	0.001996193	0.35487088	1.6027989
Gender of Child	Undernutrition (CIAF)	0.0030865	0.00075058	0.004454213	0.00040982	0.69294035	7.53135789
Dietary diversity	Undernutrition (CIAF)	0.00269552	0.00070746	0.030299061	−0.000357906	0.08896375	7.53135789
Child diarrhea	Region	0.00252383	0.00204875	0.005417416	0.00126324	0.4658741	1.99790467
Child age	Undernutrition (CIAF)	0.00215057	0.00036425	0.00050285	0.000285548	4.27675865	7.53135789
Women employment	Sanitation	0.00184471	0.00415684	0.005198262	0.003463055	0.35487088	0.53268327
Child age	Mother BMI	0.00151557	0.00024953	0.000354373	0.000192555	4.27675865	7.87082745
Mother BMI	Undernutrition (CIAF)	0.00148339	0.00019262	0.000188466	−0.000196961	7.87082745	7.53135789
Mother educational level	Water	0.00134705	0.00158335	0.00110197	0.002811513	1.22240465	0.47912053
Birth intervals	Undernutrition (CIAF)	0.00114024	0.00023401	0.000515085	0.000151399	2.21369247	7.53135789
Place of Residence	Water	0.0009086	0.00155838	0.001322628	0.001896394	0.68696676	0.47912053
Women employment	Water	0.00059892	0.00143628	0.001687725	0.00125005	0.35487088	0.47912053
Women employment	Birth intervals	0.00134705	0.00158335	0.00110197	0.002811513	0.35487088	2.21369247
Place of Residence	Undernutrition (CIAF)	0.00114024	0.00023401	0.000515085	0.000151399	0.68696676	7.53135789
Women employment	Undernutrition (CIAF)	0.0009086	0.00155838	0.001322628	−0.001896394	0.35487088	7.53135789
Women employment	Mother BMI	0.00059892	0.00143628	0.001687725	0.00125005	0.35487088	7.87082745

Authors' estimations.

The negative correlation between maternal education levels and childhood malnutrition highlights the important role of caregiver knowledge on nutrition outcomes. Environmental improvements — especially in water quality and sanitation infrastructure — are another main tool for the prevention of nutrient deficiencies. Although literature links maternal nutrition to child health, our SL-BN results indicate maternal BMI has a very weak negative association with child malnutrition (directional dependence = −0.000197). Household wealth, maternal education, and environmental conditions play more substantial roles in determining child nutritional status. But there are few risk factors that are major contributors determining malnutrition. These consist of descriptors of kids (sex, age, and also birth interval), geographical determinants (regional variations vs. urban/rural house). In addition, childhood episodes of diarrhea are a particularly important health indicator that also shape nutritional status. All of these collective factors result in a disproportionate burden among specific pediatric sub-populations within Pakistan. Our results draw attention to the need for re-thinking childhood undernourishment not as a bi-dimensional issue but, in its holistic sense: with child-level determinants and socio-economic contexts intertwined with maternal characteristics coupled with environmental risk factors in more than just additive but dynamic pathways that determine nutritional status.

It indeed fills an important gap by modeling and visualizing the underlying micro level conditional associations in child malnutrition cycle in Pakistan using structural learning – based Bayesian network. The main contribution of this article therefore is that maternal employment in households above the high-income threshold lowers the risk of undernutrition. In addition, the analysis demonstrates that the marginal effects of household wealth on nutritional outcomes declines with increasing levels of maternal education. Especially maternal schooling has particularly had a strong negative probabilistic association, as things like maternal higher educational attainment among working mothers also increases the likelihood of them implementing effective feeding practices for their infants.

Bayesian network analysis revealed a weak negative association between maternal BMI and child malnutrition (directional dependence = −0.000197, MI = 0.00148). Higher maternal BMI slightly reduces child malnutrition probability, but this extremely weak dependence provides limited independent explanatory power in the model. This study highlights the need to address maternal nutrition when developing interventions targeting child health, especially in households with differing economic status. The structure of the Bayesian network further demonstrated that positive financial status is conditionally associated with lower child malnutrition, owing to better environmental conditions like access to clean water and sanitation. Taken together, these findings highlight individual micro-level pathways through which socio-economic, maternal and environment-specific factors can shape child nutrition.

#### Output of robust test- multivaraible logistic regression

Table 5 provides multivariable logistic regression with 10-fold cross-validation estimates illuminating primary predictors of child malnutrition (Composite Index of Anthropometric Failure; CIAF) in Pakistan. The analysis at this stage indicates a troubling pattern: The risk of malnutrition increases with age and birth interval — in other words, despite the fact that larger families, on average, have better access to nutritional resources, the incidence of malnourishment is additive within those higher birth intervals. In particular, living in the city has been seen to have substantially lower odds of malnutrition than those who live in rural areas (OR = 0.78). The burden of malnutrition is not uniform; specifically, children living in Sindh (OR = 2.06), Balochistan (OR = 2.53), Khyber Pakhtunkhwa (OR = 1.28) and FATA (OR = 1.54) show deviant frequencies for malnutrition. These regions also have a much higher risk of malnutrition than the national average. Maternal nutritional status is also a key determinant; mothers with normal Body Mass Index (BMI) have lower odds of malnutrition in children (OR = 0.79). In particular, a recent history of diarrhea is positively associated with for malnutrition (OR = 1.27). Maternal education has lower odd values which means lower the risk of outcome variable, with secondary education (OR = 0.76) and higher education (OR = 0.52) associated to a lower risk of being malnourished. Interestingly, maternal employment has positive association with malnutrition (OR = 1.29), which needs further investigation as it may pertain to complex interactions among various socio-economic factors. Access to upgraded water sources (OR = 0.78) as well as sanitation facilities (OR = 0.58) consistently negatively associated which means it reduces risk of malnutrition; and households in the wealthiest quintile had the lowest odds (OR = 0.31). Finally, lower dietary diversity (less than five food items) is an individual risk factor for malnutrition (OR = 1.18; 95% CI: 1.06–1.55). The relationships that emerge in these results highlight the complex relationship between socio-demographic, environmental and economic determinants driving child nutritional outcomes in Pakistan.

**Table 5:** Output of robust test – multivariable logistic regression with cross-validation.

Factors/indicators	Odd ratios (*P*-Value)	Intervals showing 95% confidence
Child's sex: female (classify and include in reference)		
15.6-7.4,-1.8498ptMale	1.046 [0.48]	(0.93–1.19)
Child's age category (in months): 0–12 months category (classify and include in reference)		
Thirteen to twenty-four months	1.59 [0.01] ^***^	(1.28–1.98)
Twenty-five to thirty-six months	2.38 [0.01] ^***^	(1.93–2.88)
Thirty-seven to forty-eight months	2.33 [0.01] ^***^	(1.88–2.84)
15.6-7.4,-1.8498ptForty-eight to sixty months	1.94 [0.01] ^***^	(1.57–2.37)
Birth intervals: ≤ 2 years (classify and include in reference)		
Three to four years	1.22 [0.00] ^***^	(1.05–1.41)
Five to seven years	1.33 [0.001] ^***^	(1.11–1.58)
15.6-7.4,-1.8498ptExceeding seven years	1.35 [0.03] ^**^	(1.02–1.83)
Residence location: rural (classify and include in reference)		
15.6-7.4,-1.8498ptUrban location	0.78 [0.002] ^***^	(0.68–0.92)
Region/Province: Punjab (classify and include in reference)		
The Sindh region/province	2.06 [0.001] ^***^	(1.66–2.55)
The Khyber Pakhtunkhwa (KPK) region/province	1.28 [0.04] ^**^	(1.04–1.65)
The Balochistan region/province	2.53 [0.001] ^***^	(1.96–3.27)
The FATA region/province	1.54 [0.004] ^***^	(1.17–2.03)
The Gilgit Baltistan (GB) region/province	1.03 [0.89]	(0.77–1.39)
The Islamabad-Capital region	0.98 [0.88]	(0.68–1.38)
15.6-7.4,-1.8498ptThe Azad Jammu and Kashmir region/province	0.91 [0.46]	(0.79–1.19)
Maternal body mass index: below 18.5 kg/m^2^, indicating underweight status (classify and include in reference)		
15.6-7.4,-1.8498pt≥ 18.5 kg/m^2^, indicating normal status	0.79 [0.03] ^**^	(0.63 – 0.99)
Recent occurrence of diarrhea in child: no (classify and include in reference)		
15.6-7.4,-1.8498ptYes	1.27 [0.007] ^***^	(1.07–1.48)
The drinking water classification: unsafe (classify and include in reference)		
15.6-7.4,-1.8498ptSafe	0.78 [0.001] ^***^	(0.67–0.96)
Sanitation facilities type: un-Improved (classify and include in reference)		
15.6-7.4,-1.8498ptStrengthened or improved	0.58 [0.001] ^***^	(0.53–0.71)
Maternal education: illiterate (classify and include in reference)		
Primary education	0.86 [0.12]	(0.69–1.05)
Secondary education	0.76 [0.005] ^***^	(0.62–0.92)
15.6-7.4,-1.8498ptHigher education	0.52 [0.001] ^***^	(0.38–0.67)
Employment status of women: unemployed (classify and include in reference)		
15.6-7.4,-1.8498ptEmployed	1.29 [0.00] ^***^	(1.046–1.59)
Household wealth index/status: lowest wealth group (classify and include in reference)		
Lower-class category	0.92 [0.48]	(0.54–1.66)
Middle-class category	0.53 [0.000] ^***^	(0.43–0.68)
Richer-class category	0.41 [0.000] ^***^	(0.33–0.54)
15.6-7.4,-1.8498ptRichest-class category	0.31 [0.000] ^***^	(0.24–0.42)
Dietary verity status – if children consuming less than five food groups (taken as Reference group)		
Children consuming less than five food groups	1.18 [0.001] ^***^	(1.06–1.55)
Model performance metrics of multivariable logistic regression's model performance		
Number of Observations = 2,052		
Accuracy = 78.24%		
Sensitivity = 79.06%		
Specificity = 77.38%		
Precision = 0.789		
F1-Score = 0.865		
ROC curve value = 0.8093		

The authors' estimations are reported as odds ratios with corresponding *p*-values indicated in parentheses, and 95% confidence intervals presented in the third column. Statistical significance is denoted using standard notation: ^***^ for *p*-values less than 1%, and ^**^ for *p*-values less than 5%.

The ROC curve shown reflects the predictive accuracy of the Logistic Regression model in determining child malnutrition, as depicted in Figure 10A. An area under the curve (AUC) of close to 1.0 reflects a strong-performing model; for the LR model, an AUC value of ~0.80 demonstrated high levels of discriminative power with an overall accuracy measure of 78.24%. Moreover, Figure 10B introduces the confusion matrix to further elucidate our model's classification prowess at a finer level of detail. Our results show that model correctly detected 421 true positive cases of malnourished children, while it was able to classify 434 as non-malnourished true negatives. It also missed 123 malnourished patients, and misclassified 115 healthy children as malnourished. These misclassifications expose fundamental limitations in the performance of the model. Although these findings confirm its applicability for predicting anthropometric undernutrition (CIAF), they also underscore the necessity of patching methodology to ameliorate error and optimize predictive power.

**Figure 10:**
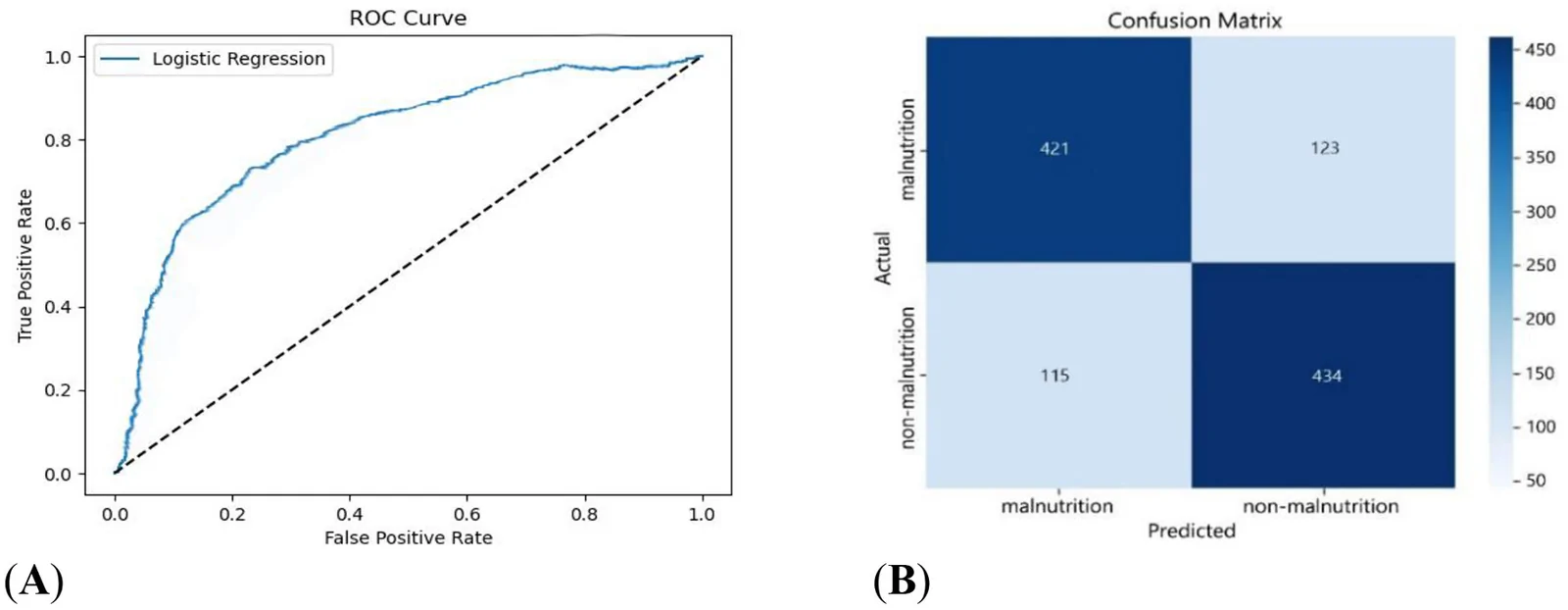
Multivariable logistic regression ROC curve and confusion matrix. **(A)** ROC Curve of Multivariable Logistic Regression. **(B)** Confusion Matrix for Multivariable Logistic Regression.

#### Micro level determinants and inequalities in child nutritional outcomes-discussion

The global rates of undernutrition in children are alarmingly high particularly in South Asia, and this was recently noted for Pakistan as well. These results are consistent with previous studies, which demonstrated that malnutrition is associated with factors such as the child's age (97–99) and birth interval (100). One more finding of our analysis supports previous studies showing negative effects of enteric pathogens on diet, but again with higher adverse impact in the poorer households, indeed specifically for diarrheal pathogens (98, 101). Having a history of these diarrheal episodes — particularly in the previous month — increases malnutrition risk 40% (2-fold) in excess of all other causes of morbidity in these vulnerable settings. It also supports the concept that poor health and nutritional outcomes among vulnerable populations are correlated along a continuum.

On the other hand, maternal factors have negative probabilistic association with on child malnutrition. Previous studies (as summarized in (102, 103)) have shown a negative link between maternal education and employment for child malnutrition (104). This association is indicative of that critical domestic role performed by mothers in terms of feeding and caring for. e.g., there is a weak negative correlation between a mother's BMI and child malnutrition. Simply put, the higher a mother's BMI is, the only slightly lower the probability of her child developing malnutrition will be, and the strength of this association is very weak. This conclusion is consistent with previous studies (105, 106). However, this impact is too small. Compared with household wealth and maternal education level, the part of the causes of child malnutrition that can be explained by the single indicator of maternal BMI alone is extremely limited, and its ability to independently explain relevant issues is not strong.

Environmental determinants have a negative association with undernutrition which accentuates the importance of Water, Sanitation and Hygiene (WASH) interventions, especially water quality and availability of sanitation facilities (81, 107). Similarly, dietary practices are also the most influential; lower odds of malnutrition are associated with higher diet diversity (11, 108). The fact that there is high heterogeneity within regions for malnutrition, particularly in rural Pakistan, is important. Such inequalities are much higher in the provinces of KPK, Balochistan and Sindh — locations where malnutrition rates have been above national averages. These spatial inequities have been previously reported (109–111) and indicate that childhood undernutrition is multidimensional, heterogeneous at local administrative units, and geographically dispersed across the country. These findings highlight the pressing need for context-relevant solutions that address the interlinked barriers to education and health access, achieving gender equity, and creating infrastructural improvements.

The analysis of Bayesian Network indicates that the most important feature concerning malnutrition is the household economics as it shows a significant negative correlation with composite anthropometric failure (CIAF). There is additional support for this relationship from logistic regression, which shows that the odds of malnutrition significantly decline across the wealth quintiles. The wealth gradient of nutrition is a clear and visible one (see Figures 11A–C showing the inverse relationship with underweight, stunting, and wasting from left to right). Unlike other demographic factors that show a steady decline across wealth quintiles, malnourishment is disproportionately found in very poor households, as determined through probability distribution analysis. This socioeconomic pattern is true for both genders, which highlights the need to factor in household resources that may drive nutritional outcomes. These mystery findings are consistent with previous research in the remaining parts of South Punjab that revealed experienced hunger sameness and wasting all these low-income children is 3 times higher as amongst children from high-income households. In addition, having the data shows a clear SES gradient for both stunting and wasting amongst these children as well (22, 112).

**Figure 11:**
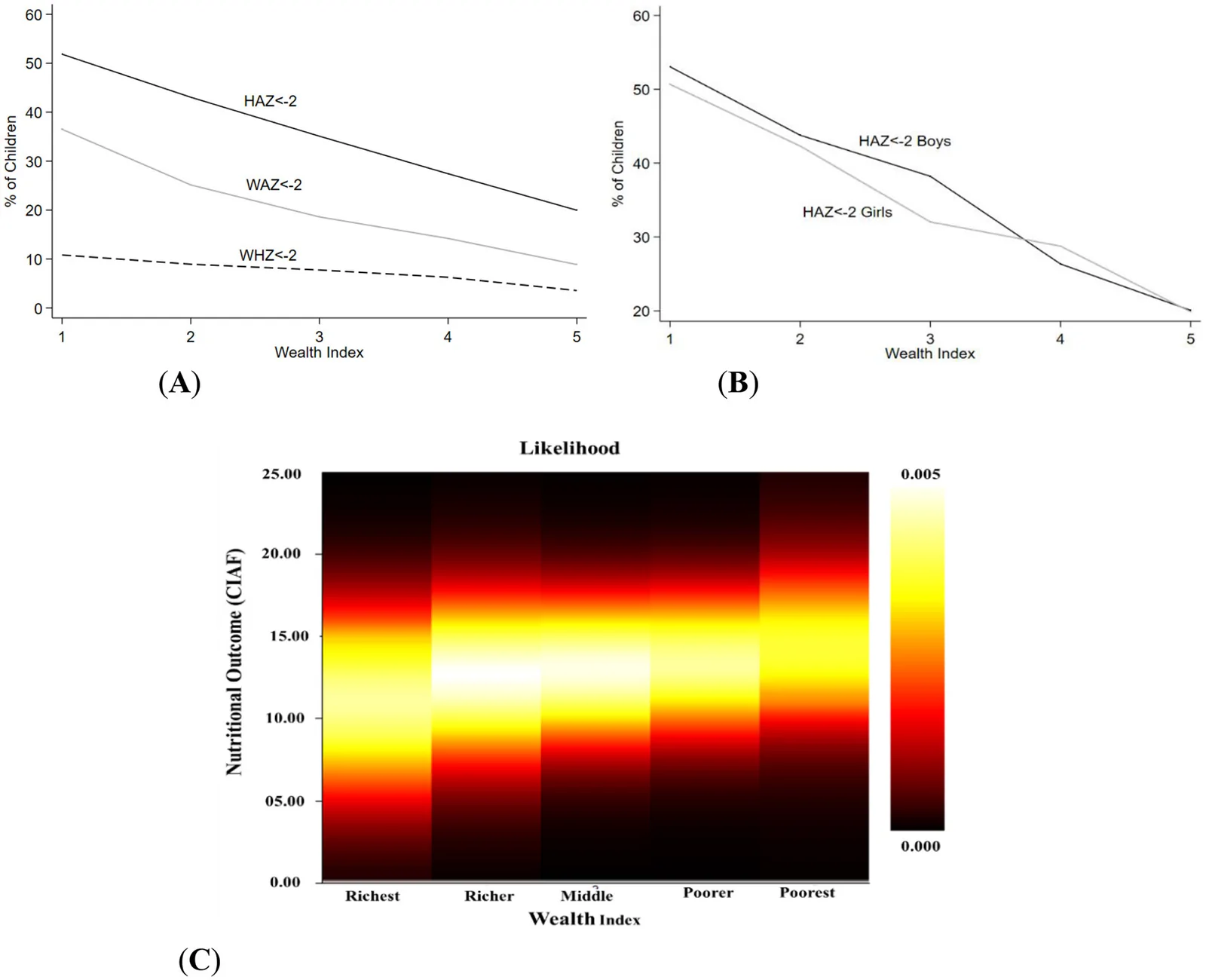
Nutritional Outcomes by Wealth Status Quintiles, By Sex. **(A)** by wealth status. **(B)** disaggregated by gender. **(C)** Joint Distribution Between CIAF vs. Wealth with Mesh Query.

Insufficient wealth or income is estimated to account for a large proportion of variation in child nutrition outcomes (113). This study shows a concentration index of −0.1727 (*p* < 0.001), indicating that child malnutrition is disproportionately concentrated among the lowest wealth quartiles (relative inequality). The concentration curve lies above the equality line (Figure 12A), further confirming this pattern. The Slope Index of Inequality (SII = −0.445, Figure 12B) estimates an approximately 44.5 percentage point absolute difference in malnutrition prevalence between the poorest and richest wealth quintiles. These findings are consistent with past studies highlighting wealth-related inequalities in undernutrition of children aged 0 to 59 months in developing countries such as Pakistan, Bangladesh, India, Nepal and Ethiopia (114–118).

**Figure 12:**
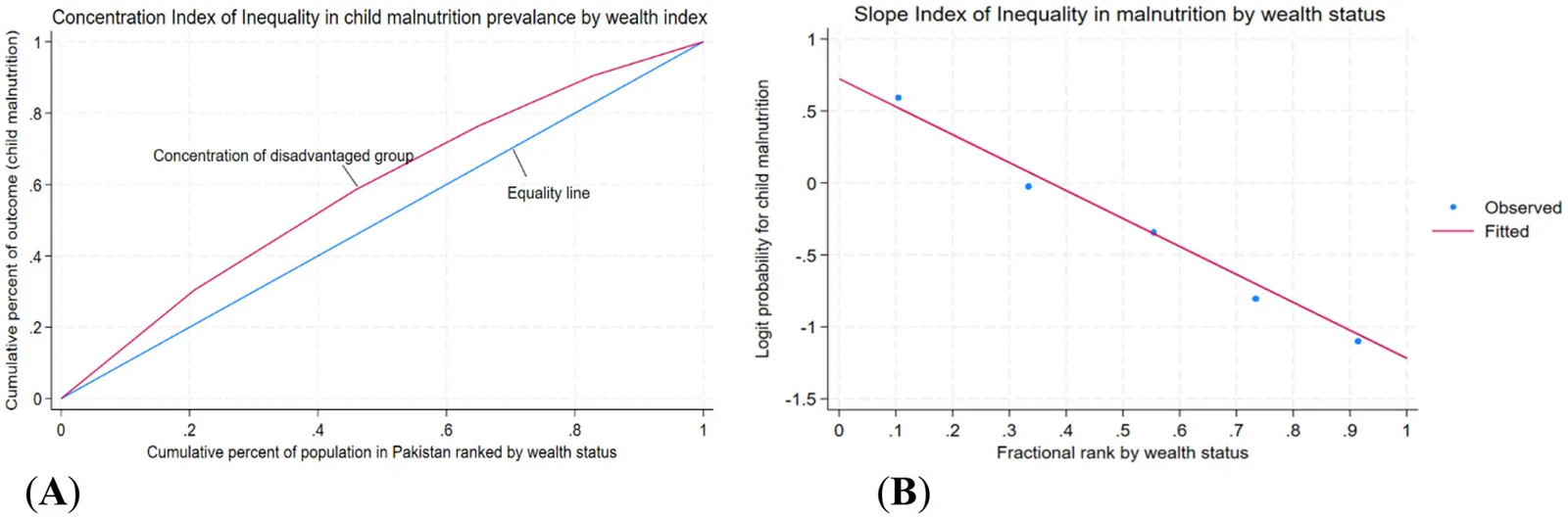
Inequity in Undernutrition between Wealth Classes. **(A)** Concentration Index for inequalities. **(B)** Slop Index of Inequality (SII).

Wealth status has been found to play an important role in regional discrepancies in nutritional outcomes according to studies conducted within Ghana, India and Bangladesh (12, 13, 119). Capitalizing on the findings provided by these pioneering studies, this article uses an equi-plot to assess absolute wealth-based inequalities in child malnutrition between provinces in Pakistan. Findings reveal regionally relevant discrepancies in malnutrition rates among children.

As shown in Figure 13, each wealth quintile is represented as a dot that extends continuously from the poor (via mid-tone) to the rich (depicted with the color yellow), while malnutrition rates within various provinces are illustrated by province region. Wider horizontal spans between such points show a greater discrepancy in geographic malnutrition. It further reveals that the wealth index the malnutrition is most significant in Sindh, Balochistan & Federally Administered Tribal Areas (FATA) —the provinces rated with highest poverty also available to Pakistan. We show the joint probability distribution of nutritional outcomes among those sets of zones here in Figure 14. The findings reveal a notably higher malnutrition burden in Sindh, Balochistan and FATA than elsewhere while it finds stronger joint distributions with wealth and lower functional indicators of malnutrition found for Punjab, ICT, GB and AJK. Within the earlier sections we also observe patterns which align with prior literature showing higher prevalence of stunting, and large wealth-based inequities in Sindh (112) and Balochistan (120–122), as well as documented regional inequities in stunting for Middle Eastern countries including Egypt, Jordan and Yemen (123).

**Figure 13:**
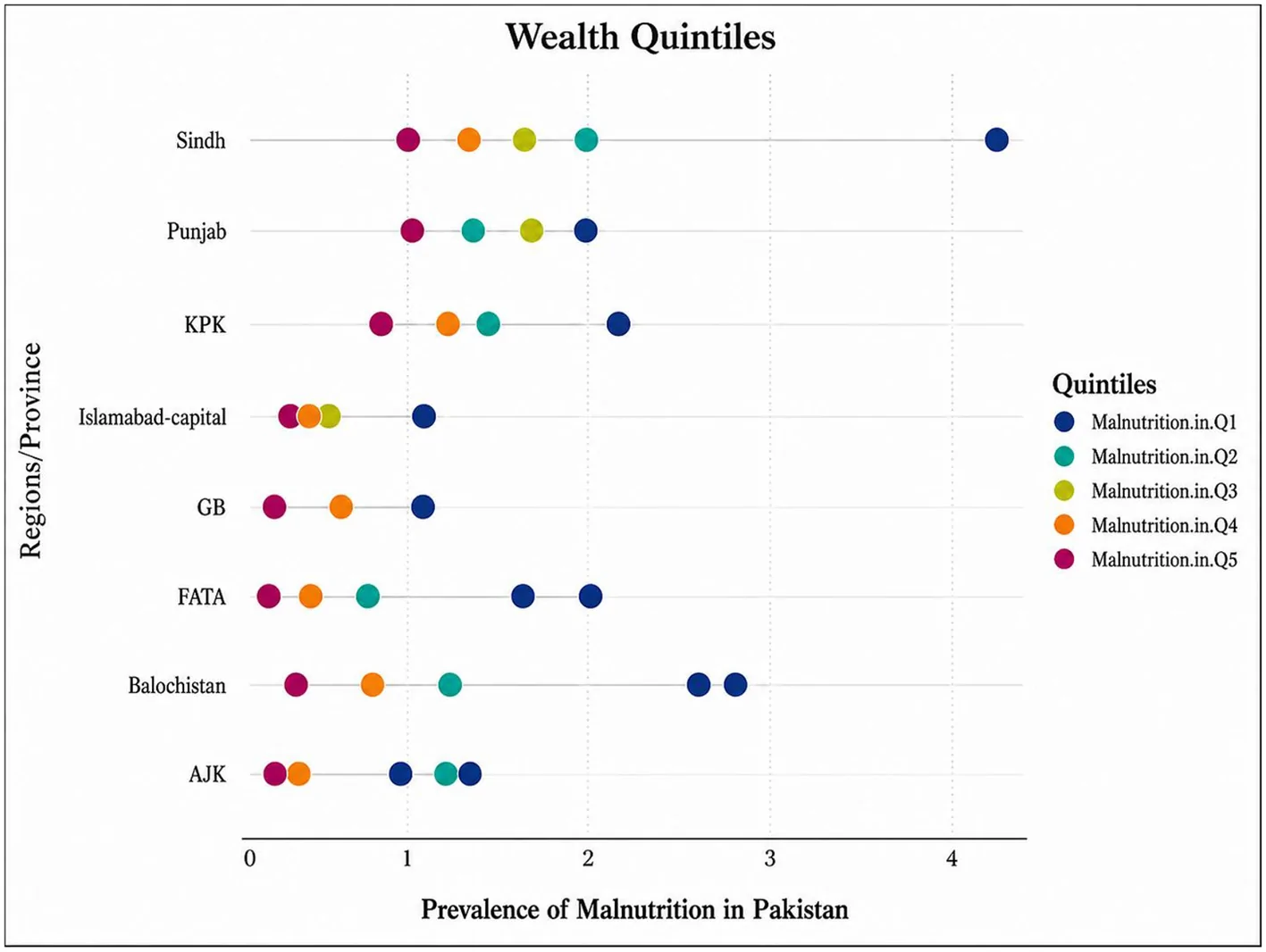
Analyzing the inequalities of undernutrition among various income groups in different region/provinces.

**Figure 14:**
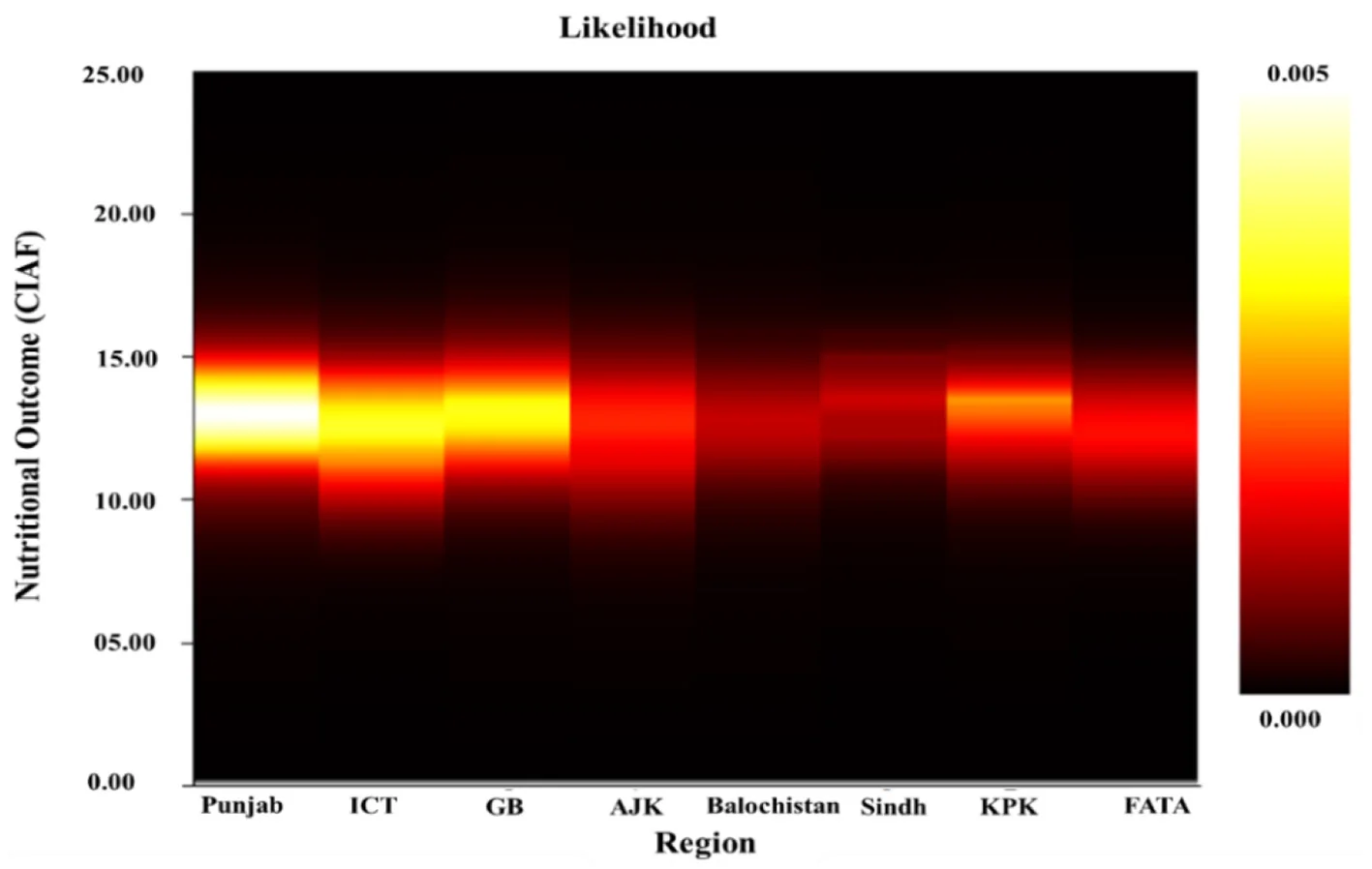
Mesh query for Joint distribution between nutritional outcome (CIAF) and region/provinces.

##### Joint probability distribution of the nutritional outcome with respect to region

Figure 14 shows the joint probability distributions for child undernutrition (CIAF) across provinces/regions. The mesh query visualization indicates that the probability masses are concentrated in the areas of highest CIAF, and Sindh, Balochistan, and FATA had also the highest probabilities of undernutrition. On the contrary, Punjab, ICT, GB and AJK have lower undernutrition probabilities with wider distributions. These changes correspond to the concentration index results shown in Table 3 and Figure 13, as well as with variation across regions. The joint distribution provides evidence that undernutrition is not randomly distributed across geographical locations in Pakistan, as the most deprived hubs clearly require geographically-focused interventions. These regional patterns are consistent with the concentration index results (Figure 12A, CI = −0.1727, *p* < 0.001) and the wealth-based inequality analysis (Figure 13), which showed that undernutrition is disproportionately concentrated among poorer populations, particularly in disadvantaged provinces. The joint distribution provides compelling evidence that undernutrition is not randomly distributed across Pakistan's geography; rather, it is systematically concentrated in the most deprived regions. This underscores the urgent need for geographically-targeted interventions that address the specific structural and socioeconomic drivers in these high-burden areas.

#### Bridging between macro and micro evidence: triangulation of policy analysis & quantitative findings

Combined qualitative policy dialogue and quantitative findings from Bayesian networks reveal a cohesive narrative that is above what can be achieved with either independently. So, by providing this triangulation on governance, aid architecture, and equity dimensions—this proposal has sought to enable a deeper understanding of the structural forces shaping child undernutrition in Pakistan, empirically grounded insights that note both the extent and the character of what needs addressing for effective policy action.

##### Governance, institutional capacity and nutrition outcomes

Policy analysis described continued fragmentation of nutrition governance in Pakistan, indicated by weak inter-sectoral coordination to ensure an integrated approach to nutrition including for agriculture issues; unstable policy frameworks guided often by short-term donor-funded projects compromising longer-term institutional capacity (27, 59). The governance gap is especially pronounced in the period after devolution, as poor horizontal coordination between provincial departments and weak vertical integration between national priorities and local implementation have hampered service delivery (60). The results obtained with the Bayesian models support our hypothesis quantitatively: public health spending and private health expenditure were two of the best negative probabilistic predictors of child undernutrition (Table 2), providing evidence that sustained domestic financing for health services is protective. Furthermore, the strong positive probabilistic associations of poverty, inflation and undernutrition (Table 2) substantiate the role that macroeconomic instability by itself or through its social protection strategies increases nutritional vulnerabilities. These converging lines of evidence imply that failures in the governance of child nutrition — rather than nutritional resources constraints alone, are key binding obstacles to improving child nutrition outcomes and whole system reforms are needed in terms of institutional coordination, accountability, and policy coherence.

##### Foreign assistance and national ownership

A Paradoxical Relationship: The policy analysis presented serious issues about Pakistan's past reliance on foreign aid to finance nutrition programming, arguing that such dependency may paradoxically undermine national ownership and institutional capacity (27, 59). Although international funding supports both critical nutrition interventions, such as community management of acute malnutrition (CMAM), vitamin A supplementation and therapeutic feeding (61, 64), it has also led to a fragmented patchwork of donor-driven programming that frequently operates independently of national health systems. This critique of the policy should be interpreted in conjunction with the quantitative finding that suggests a negative relationship between external health expenditure and undernutrition (Table 2). This link could reflect the availability of life-saving interventions to meet acute needs but does not in itself mean that foreign aid builds institutional capacity for sustainable food production and long-term nutrition security. As discussed earlier, qualitative evidence does appear to suggest aid-dependent programming may perpetuate fragmentation of services, and distract resources from systemic strengthening whilst creating unsustainable service delivery infrastructures which rely on continued external support (79, 80). The overlap of these perspectives suggests that foreign assistance can be well-directed and effective when it is aligned with national priorities, where resources are integrated directly into existing health systems but may inadvertently undermine institutional capacity when operating outside the framework of functioning national environments.

##### Inequity, social protection and structural exclusion

The analysis pointed within policy analysis that in Pakistan social protection programs especially the Benazir income support program (BISP) has been deprived by corruption, political patronage and administrative inefficiencies leading to systematic exclusion of the poorest population being excluded for coverage under safety nets (69, 72, 75, 76). These concerns around equity receive strong empirical support from the quantitative findings. Substantial socioeconomic inequality was observed. The negative concentration index (CI = −0.1727, *p* < 0.001) indicates that undernutrition is disproportionately concentrated among poorer households (relative inequality). The Slope Index of Inequality (SII = −0.445) further shows an absolute gradient, suggesting an approximately 44.5 percentage point difference in malnutrition prevalence between the poorest and richest wealth quintiles (Figure 12). The disparity is even greater when comparing the provinces, with malnutrition concentrated disproportionately among the most impoverished provinces where good governance capacity is at its lowest and social protection coverage is also weak (Figures 13, 14) (112, 120–122). However, the qualitative and quantitative evidence (113–118) identified above of persistent inequality layers with concerns about programmatic failure to reaffirm a compelling policy argument: without systematic reforms in targeting, accountability, and coverage of social protection programs, undernutrition will continue to be disproportionately felt by the poorest and marginalized.

##### Synthesis of evidence: implications for policy

The synthesis of qualitative and quantitative evidence indicates that child undernutrition in Pakistan is not just a health or nutrition issue, but ultimately a governance problem embedded within wider structural inequalities. These three converging trends (institutional fragility, aid dependence, inequitable social protection) are mutually reinforcing: poor governance leads to ineffective delivery of nutrition services, the continuing reliance on aid sustains discontinuous programming and inequitable social protection results in those most needy not receiving support. This cycle can only be broken by a comprehensive, multi-sectoral solution addressing both the immediate determinants (dietary diversity, maternal education, water and sanitation) and underlying structural factors (poverty, inflation, governance capacity, social protection systems). The triangulation of evidence put forth here gives a strong empirical basis to such an approach, and highlights the need for immediate cross-sectoral and multi-governance action.

## Conclusion

This study is mainly concerned with depicting how multiple factors, especially governance and policies at the macro and micro levels, as well as socio-economic factors for individuals, affect child health and create inequalities in Pakistan. For this purpose, at the macro level, the study critically discussed the history of nutritional policies and programs in Pakistan. Moreover, this study also used World Development Indicators data and UNDP Human Development Index data to assess the impact of macro-level indicators that affect children's undernutrition. At the micro level, this study assessed the impact of socio-economic factors that affect the nutritional outcomes of children and create inequalities in malnutrition in Pakistan using demographic and health Survey data (PDHS-2017-18). The study employed the probabilistic SL-BN to deeply understand the strength of association and conditional dependencies among macro and micro indicators and child undernutrition in Pakistan. Moreover, the study also performs robust analysis for micro-level data by employing logistic regression and concentration index for inequalities measurement by wealth status.

The undernutrition in all its forms among under-five children in Pakistan is 44.15%. Stunting, underweight, and wasting rates are 38.13%, 23.04%, and 8.05%, respectively. The logistic results almost proved supportive of intelligent networks. The finding of score-based and constrained-based learning approaches of probabilistic SL-BNs analysis at the macro level depicted probabilistic relationship with child malnutrition. inflation (MI = 0.6234) and poverty (MI = 0.6538) found the strong positive probabilistic associations. Furthermoe, Income (MI = 0.1416), HDI (MI = 0.1331), and private health spending (MI = 0.1208) found a weak-to-moderate negative probabilistic associations. While the Government health spending (MI = 0.0003) and foreign health dependency (MI = 0.0044) depicted negligible associations. These macro-level findings are exploratory due to the limited time-series (*n* = 23) and do not imply causal effects. Similarly, at the micro level, the intelligent probabilistic Bayesian network found that child malnutrition has a solid negative probabilistic relationship with dietary diversity, wealth status, maternal employment, mother education, mother BMI, and environmental factors, which showed that increase in the probability of these factors would reduce the probability of child malnutrition at the individual or micro level in Pakistan. Conversely, the score-based and constrained-based learning approach of probabilistic SL-BN found that child malnutrition has a solid positive probabilistic relationship with children of higher age, child higher birth interval, disadvantaged regions, rural places of residence, and children have diarrhea, which showed that increasing the probability of these factors will are positively associated with child undernutrition at the individual level in Pakistan.

Simultaneously, this study revealed an important novel finding at both macro and micro-levels. At both scales of the analyses, a structural Bayesian Network (SL-BN) indicated that the random variables contained in network structure are jointly distributed and conditionally dependent. The aggregate context of learning on the SL-BN led to constraint-based determination that income (with or without integrated spending) conditionally decreases by probability, the predictor of child hunger, with prior percentage marginal increase effect from Human Development Index. Moreover, the results of Bayesian network analysis revealed that inflation increases the chances of malnutritional conditional on controlling percentage effect of poverty at macro level in Pakistan.

At the micro level, the probabilistic SL-BN detected conditional effects of higher household wealth status and maternal education that reduced child malnutrition; this result accounts for mothers who are employed as a percentage effect in Pakistan. Additionally, the Bayesian networks revealed that greater maternal education, better maternal BMI and good water and sanitation are other important environmental factors that conditionally reduced the individual-level probabilities of child malnutrition about household wealth status percentage effect in Pakistan.

At micro-level, the concentration index results indicate that child undernutrition is disproportionately high among the poorest wealth groups. Sindh, Balochistan, FATA and KPK (i.e., some of the most underprivileged regions in Pakistan) have extremely lower absolute inequality scores than many other developed areas of Pakistan. As a result, nationally the difference in child undernutrition levels between richest and poorest wealth quintiles is an alarming 44.5%.

Furthermore, this study's empirical results are distinct in terms of its theme and approach (SL-BN), distinguishing it from earlier research and adding to its significance. Overall, this study concludes that poor socio-economic conditions of individuals affect nutritional outcomes and create inequalities at the micro level, which reflects the poor performance of macro indicators and health status at the national level. In other words, poor nutritional policies and programs and poor government performance at the macro level are associated with health status and inequalities at the micro level. The critical discussion on nutritional policies and programs highlighted that the government of Pakistan always lacked a concrete strategy to combat malnutrition as Pakistan has only relied on the history of short-term solutions regarding nutritional programs mainly funded or aided by United Nations agencies (UNICEF, WFP, FAO, & WHO) or non-government organizations (NGOs). Although in the short-term, foreign aid can be vital for combating malnutrition, in the long-term, it can prove a curse for Pakistan.

### Practical implications of this study

This study's findings highlight the pressing necessity for Pakistan to implement a comprehensive, long-term, and nationally coordinated strategy to address child undernutrition. At the macro level, these findings highlight the critical role of continued investment in human development, domestic health financing and policies for price stability and poverty control. An overreliance on temporary, donor-funded initiatives has proven to be both inadequate and unsustainable. Therefore, national design policies domestically-based nutrition should become a priority of policy makers, reinforced through strong political will in the public sector and improved capacity institutions.

Microscopically, even at the individual child level who presents to the Pakistan Pediatric Emergency Unit (PPEU) there is an urgent need for tailored interventions and targeted programs given that nutritional targets are not uniform nationwide, with wide variation across regions and underprivileged provinces such as Sindh, Balochistan, FATA & KPK. This highlights the immediate need for programs to educate mothers and promote dietary diversity; provide access to clean tap water, sanitation facilities and social protection for poor households. Here we present a proof-of-concept framework using structural learning-based Bayesian networks (SL-BNs) to identify high-impact variables and conditional dependencies, which could enable more targeted evidence-based policymaking in this field. By pursuing a two—pronged strategy of macro-economic progress at the national level and closing inequities gaps at the grassroots, Pakistan can tap into this demographic dividend and ensure children are given an equal start in life to enable them all become productive members of their workforce, reducing childhood undernutrition rates significantly while promoting social wellbeing.

### Strengths, limitations, and future research directions

Hence, in this study we analyze the macro- and micro-determinants of child undernutrition in Pakistan through a complete Bayesian network (BN) algorithm. The SL-BN is a probabilistic model, and its probabilistic output can be beneficial to describe the joint probabilities for areas conditioned by multiple different sources. The combination of SL-BN approaches with conventional analytics like logistic regression and the concentration index augments our results in terms of robustness. This work is the first to collectively review how macroeconomic nutritional governance constructs lead to suboptimal individual nutritional outcomes on a wider scale and highlight the need for improvements in both sectors as potential mechanisms to facilitate effective alignment across governance levels. By jointly examining macro-level structural conditions and micro-level household determinants, this study fills a gap in the existing academic literature of Pakistan. It not only establishes a starting framework for subsequent research in the same field, but also lays a solid foundation for the practical application work that needs to be implemented.

This study has one limitation: the listwise deletion method was adopted when processing missing data. In simple terms, this method means that as long as any piece of information in a set of data is missing, all other valid data in that set will be deleted along with the entire row, and none of them can be used. The proportion of missing values for each individual variable collected in the study was lower than 3%, meaning the amount of missing data itself was very small. However, due to adhering to the criterion that “all data must be complete to be included in the group for analysis,” the final sample size available for statistical analysis was directly reduced from the original 4,499 to only 2052. Subsequently, the study conducted a specific sensitivity analysis. For comparison, one group consisted of the samples with only complete data available, and the other group consisted of all original collected samples. After comparison, there was no significant difference in the core characteristics of the two groups of samples, with the corresponding *p*-value greater than 0.10. No regular systematic differences are observed in the measurable characteristics across groups—put plainly, these directly observable and quantifiable features of the research subjects do not exhibit clear-cut disparities between the compared populations, where one group universally possesses a trait that the other group universally lacks. However, non-significant trials do not rule out selection bias rising from unobserved characteristics. We consequently recognize possible non-random missingness as a limitation of the complete-case investigation. This study used 10-fold cross-validation to conduct internal validation for the applied logistic regression model. By internal validation, we mean that we only used the dataset on hand for this study to test whether the model can function properly within this dataset. However, this internal evaluation that only works on our own dataset cannot guarantee that the model will perform equally well when applied to other completely different scenarios. For future relevant research, it is necessary to use independent datasets from other regions or different survey waves—that is, brand-new data completely unrelated to this study—to conduct external validation once again. The goal of this external validation is to confirm that the various influencing factors identified in this study can remain stably valid across different settings. The listwise deletion method we use refers to a data processing approach where an entire entry of data is completely excluded from use if any portion of the content is missing from that single entry. Although this method is indispensable, its application will reduce the power of statistical tests—simply put, it weakens the ability to use statistical methods to verify research conclusions, making it harder for analyses to detect originally truly existing correlations or effects. For future related research, researchers may try multiple imputation or full information maximum likelihood to assess the sensitivity of network models to assumptions related to missing data; that is, to test whether the network model used in a given study will produce different results due to different assumptions about missing data, and measure its level of sensitivity to those assumptions.

However, other limitations include the cross-sectional nature of the microdata and a tendency to generalize regionally. Moreover, inequality analysis is limited to microdata; the insights provided by macro-level inequality metrics are ignored. Future studies should use longitudinal or geospatial methods to evaluate the short-term impacts of particular policy interventions.

## Data Availability

This study utilized secondary data from the Pakistan Demographic and Health Survey 2017 -18, UNDP, and World Bank databases. The data are freely accessible online at the following links: PDHS: https://dhsprogram.com/data/dataset/Pakistan_Standard-DHS_2017.cfm?flag=1. UNDP: https://hdr.undp.org/data-center/human-development-index#/indicies/HDI. World Bank WDI: https://databank.worldbank.org/source/world-development-indicators. The STATA do file and Python codes are available in HTML format through the corresponding author upon reasonable request.

## Footnotes

1 Scutari M, Denis JB. Bayesian Networks: With Examples in R. Boca Raton: CRC Press (2014). Online available at: https://books.google.be/books?hl=en&lr=&id=v-XMAwAAQBAJ&oi=fnd&pg=PP1&dq=Scutari,+M.,+%26+Denis,+J.-B.+(2014).+Bayesian+Networks:+With+Examples+in+R.&ots=AJkciFhtXm&sig=VjZWHiu7Z3aJy64hQquLpg2yMdM&redir_esc=y#v=onepage&q&f=false.

2 Fairclough N. Critical discourse analysis: The critical study of language. Abingdon: Routledge (2013). Available at: https://books.google.be/books?hl=en&lr=&id=3djbAAAAQBAJ&oi=fnd&pg=PT6&dq=Fairclough,+N.+(1995).+Critical+discourse+analysis:+The+critical+study+of+language.+London:+Longman.&ots=ITeU26cih4&sig=7BDsOpsBqsrdH9O8iCyDKfRmbUA&redir_esc=y#v=onepage&q=Fairclough%2C%20N.%20(1995).%20Critical%20discourse%20analysis%3A%20The%20critical%20study%20of%20language.%20London%3A%20Longman.&f=false (Accessed August 13, 2026).
