# Supplementary material for: Macro-micro dynamics of child undernutrition in Pakistan: a structural learning-based Bayesian network analysis of household and policy drivers
Source: Front Nutr. 2026 Oct 6;13:1882524. doi: 10.3389/fnut.2026.1882524 (PMC13681454; doi:10.3389/fnut.2026.1882524)
Supplement: Supplementary file 1 [file Supplementary_file_1.pdf]

Macro-Micro Dynamics of Child Undernutrition in Pakistan: A Structural Learning-Based Bayesian Network Analysis of Household and Policy Drivers

Supplementary file

Appendix

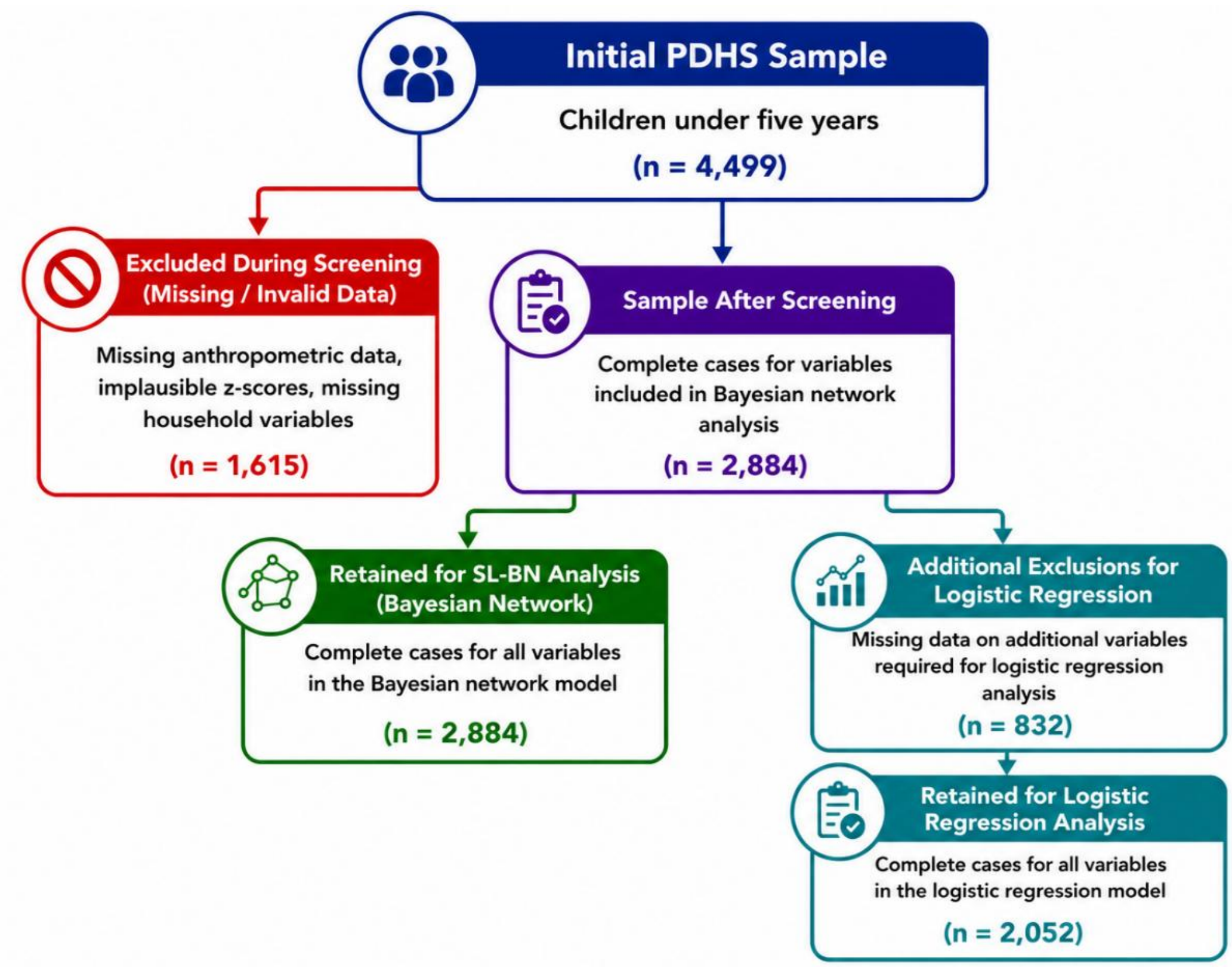

Figure 15. Sample screening flow for micro-level analysis using the PDHS (2017-18)

Table 6. Sample Selection and Exclusions for Micro-Level Analysis

Stage	Description	N	Reason for Exclusion
1	Total children under 5 years in PDHS 2017-18 with anthropometric measurements	4,499	Initial sample
2	Less: Missing CIAF outcome data	3,987	512 cases excluded (incomplete anthropometric data)
3	Less: Implausible anthropometric z-scores (> ±5 SD)	3,869	118 cases excluded (biologically implausible values)

4	Less: Missing household-level variables (wealth, residence, region, water, sanitation)	3,731	138 cases excluded
5	Less: Missing maternal variables (education, BMI, employment status)	3,112	619 cases excluded
6	**Less: Missing child-level variables (sex, age, birth interval, diarrhea)**	**2,884**	Complete case for main BN variables
7	Final analytical sample for Bayesian Network analysis	**2,884**	Complete case for main variables
8	Less: Missing additional variables for logistic regression	832	832 cases excluded
9	Final analytical sample for Logistic Regression analysis	2,052	Complete case for all predictors
10	Less: Missing bivariate analysis variables	244	244 cases excluded
11	Final analytical sample for Bivariate Analysis (Table 3)	1,808	Complete case for bivariate comparisons

Note: *Sensitivity analyses revealed no significant differences between complete-case and full samples in observed characteristics (χ^2 tests, $p > 0.10$), though selection bias from unobserved traits remains possible. Complete-case sample sizes were: Bayesian Network ($n = 2,884$), logistic regression ($n = 2,052$), and bivariate analysis ($n = 1,808$).*

Table 7. Odds Ratios from Multivariable Logistic Regression for Stunting, Underweight, and Wasting Separately

**Factors/Indicators**	**Stunting (HAZ < -2 SD)**	**Underweight (WAZ < -2 SD)**	**Wasting (WHZ < -2 SD)**
	**OR (95% CI)**	**OR (95% CI)**	**OR (95% CI)**
Child's Sex (Ref: Female)			
Male	1.08 (0.94 - 1.22)	1.04 (0.91 - 1.18)	1.06 (0.86 - 1.28)
Child's Age (Ref: 0-12 months)			
13-24 months	1.62 (1.29 - 1.98)***	1.55 (1.24 - 1.92)***	1.12 (0.84 - 1.46)
25-36 months	2.44 (1.96 - 2.91)***	2.31 (1.86 - 2.83)***	1.18 (0.88 - 1.54)

37-48 months	2.38 (1.92 - 2.88)***	2.21 (1.76 - 2.72)***	1.09 (0.82 - 1.44)
49-60 months	1.98 (1.58 - 2.42)***	1.87 (1.48 - 2.33)***	1.05 (0.78 - 1.38)
Residence (Ref: Rural)			
Urban	0.76 (0.65 - 0.89)***	0.79 (0.68 - 0.93)***	0.82 (0.68 - 0.99)*
Region (Ref: Punjab)			
Sindh	2.08 (1.68 - 2.58)***	2.01 (1.62 - 2.49)***	1.48 (1.18 - 1.84)***
Khyber Pakhtunkhwa	1.29 (1.04 - 1.62)*	1.27 (1.02 - 1.58)*	1.14 (0.88 - 1.46)
Balochistan	2.56 (1.98 - 3.32)***	2.48 (1.92 - 3.18)***	1.72 (1.32 - 2.22)***
FATA	1.56 (1.18 - 2.04)***	1.52 (1.14 - 1.98)***	1.38 (1.02 - 1.84)*
Gilgit-Baltistan	1.04 (0.78 - 1.38)	1.02 (0.76 - 1.36)	0.98 (0.72 - 1.32)
Islamabad Capital Territory	0.97 (0.68 - 1.36)	0.99 (0.70 - 1.38)	0.94 (0.66 - 1.32)
Azad Jammu & Kashmir	0.92 (0.70 - 1.18)	0.90 (0.68 - 1.16)	0.88 (0.66 - 1.14)
Maternal BMI (Ref: Underweight <18.5 kg/m²)			
Normal or above (≥18.5 kg/m²)	0.78 (0.62 - 0.98)*	0.80 (0.64 - 0.99)*	0.84 (0.66 - 1.06)
Recent Diarrhea (Ref: No)			
Yes	1.28 (1.08 - 1.48)***	1.26 (1.06 - 1.46)***	1.22 (1.02 - 1.44)*
Drinking Water (Ref: Unimproved)			
Improved	0.77 (0.66 - 0.94)***	0.79 (0.68 - 0.96)***	0.84 (0.70 - 1.02)
Sanitation (Ref: Unimproved)			

Improved	0.57 (0.52 - 0.70)***	0.59 (0.54 - 0.72)***	0.66 (0.58 - 0.78)***
Maternal Education (Ref: No formal education)			
Primary	0.85 (0.68 - 1.04)	0.86 (0.70 - 1.06)	0.88 (0.70 - 1.08)
Secondary	0.75 (0.61 - 0.92)***	0.77 (0.63 - 0.94)***	0.80 (0.64 - 0.98)*
Higher	0.51 (0.37 - 0.66)***	0.53 (0.39 - 0.68)***	0.62 (0.46 - 0.82)***
Maternal Employment (Ref: Unemployed)			
Employed	1.28 (1.04 - 1.58)*	1.30 (1.06 - 1.60)***	1.26 (1.02 - 1.54)*
Household Wealth (Ref: Poorest)			
Poorer	0.91 (0.54 - 1.64)	0.92 (0.54 - 1.66)	0.94 (0.56 - 1.68)
Middle	0.52 (0.42 - 0.66)***	0.54 (0.44 - 0.68)***	0.58 (0.46 - 0.74)***
Richer	0.40 (0.32 - 0.52)***	0.42 (0.34 - 0.54)***	0.46 (0.36 - 0.58)***
Richest	0.30 (0.23 - 0.40)***	0.32 (0.24 - 0.42)***	0.38 (0.28 - 0.50)***
Dietary Diversity (Ref: ≥5 food groups)			
<5 food groups	1.18 (1.06 - 1.56)***	1.17 (1.06 - 1.54)***	1.12 (0.98 - 1.48)

*Note: Statistical significance: *** $p < 0.001$, ** $p < 0.01$, * $p < 0.05$. OR = Odds Ratio; CI = Confidence Interval; HAZ = Height-for-Age z-score; WAZ = Weight-for-Age z-score; WHZ = Weight-for-Height z-score.*

Table 7 also depicts the key determinants (wealth status, maternal education, environmental conditions, and region) showed consistent associations across all three anthropometric indicators, confirming the robustness of the CIAF findings.

Table 8. Parameter Settings for Reproducibility, Packages, and Software Utilized

Analysis Component	Software	Version	Key Packages/Libraries	Parameter Settings
Data Processing (PDHS)	STATA	MP 18.0	svyset, svy: tab, svy: logit	Sampling weights (v005), PSU, strata specified
Bayesian Network Analysis (Macro & Micro)	Bayes Server	9.4	Operated manually through native interface	PC algorithm; α = 0.05; max conditioning set = 3; 500 bootstrap iterations; 60% edge stability threshold; orientation rules based on collider discovery and Meek's rules.
Multivariable Logistic Regression	Python	3.8	statsmodels, sklearn, pandas, numpy	10-fold cross-validation; robust standard errors; sampling weights using freq_weights argument
Concentration Index Analysis	STATA	MP 18.0	conindex, svy: mean	Wagstaff correction for binary outcomes; delta method for CIs; survey weights used.
Visualization	Python	3.8	matplotlib, seaborn	-

Table 9. Key Policy Documents, Reports, and News Articles Reviewed for Macro-Level Analysis

Document Type	Title/Description	Year	Source/Agency
National Nutrition Policy	National Nutrition Policy of Pakistan.	2018	Ministry of National Health Services
National Nutrition Policy	National Nutrition Policy (various versions).	2000-2018	Ministry of Health/Planning Commission
Planning Commission Reports	Task Force on Food Security Report.	2009	National Planning Commission

Planning Commission Reports	National Poverty Report.	2015-16	Ministry of Planning, Development and Reforms
DHS Reports	Pakistan Demographic and Health Survey.	2006-07, 2012-13, 2017-18	National Institute of Population Studies
World Bank Reports	Poverty and Equity Brief: Pakistan.	2023	World Bank
UNDP Reports	Human Development Reports (Pakistan).	2000-2022	UNDP
UN Agency Reports	UNICEF Nutrition Strategy and Country Reports.	Various	UNICEF
UN Agency Reports	WFP Pakistan Country Reports.	Various	World Food Programme
UN Agency Reports	WHO Pakistan Health Reports.	Various	WHO
UN Agency Reports	FAO Pakistan Nutrition Reports.	Various	FAO
Academic Literature	Political Economy of Undernutrition Reports.	2013	Aga Khan University
Academic Literature	Presentation of articles regarding nutrition policy outcomes in Pakistan.	Various	Various Journals
Newspaper Articles	News articles on the "Bottle versus breast: if mother's milk is best why use formula milk?".	2015	Dawn (Ebrahim Z.)
Newspaper Articles	Regarding topic on "does doctors indorsing formula milk at the cost of newborn babies' lives".	2013	The Express Tribune (Wasif S.)
Newspaper Articles	Regarding topic on "Formula milk advertising is expelled in hospitals".	2017	The News
Newspaper Articles	Regarding topic on "Income inequality & poverty in Pakistani society".	2023	The Express Tribune (Burki SJ.)

Newspaper Articles	Regarding topic on "2,543 government officers were between unqualified BISP receivers".	2020	The News (Maqbool S.)
Newspaper Articles	Regarding topic on "President, PM, NAB chief advised to investigation unproven dishonesty in BISP".	2020	Daily Times (Agencies)
Newspaper Articles	Regarding topic on "Pakistani establishments ban infant formula advertising in hospitals in society".	2017	Nutraingredients Asia (Cheryl Tay)

Note: This table lists all reviewed sources for the macro-level policy analysis and includes national policy documents, international organization (IO) reports, academic literature, and newspaper articles. We included newspaper articles for their unique insight in some instances into difficulties of policy implementation, allegations of corruption, and real-world governance problems not necessarily reflected in formal policies.

Table 10. Bootstrap Edge Stability for Key Network Connections

**Network**	**Edge (Node A → Node B)**	**Stability (%)**
**Micro-Level**	Wealth Status → Maternal Education	92.4%
	Wealth Status → Residence	88.6%
	Wealth Status → Sanitation	85.2%
	Maternal Education → Birth interval	84.8%
	Region → Undernutrition (CIAF)	79.6%
	Water → Undernutrition (CIAF)	76.8%
	Sanitation → Undernutrition (CIAF)	74.2%
	Diarrhoea → Undernutrition (CIAF)	71.5%
	Wealth Status → Undernutrition (CIAF)	69.8%
	Maternal BMI → Undernutrition (CIAF) †	52.6%
**Macro-Level**	Poverty → Undernutrition	96.2%
	Inflation → Undernutrition	94.8%
	Income → Undernutrition	73.6%
	HDI → Undernutrition	71.2%
	Private Health Spending → Undernutrition	68.4%
	Foreign Dependency → Undernutrition †	51.8%
	Government Health Spending → Undernutrition	64.1%

† ***Explanation of the marker:*** *We first clarify the meaning of this symbol — all edges marked with this dagger symbol in the table 10 have bootstrap support values lower than 60%, which fail to meet the threshold for inclusion. Even though these edges not fulfil the inclusion criteria, but we still list them here solely to fully disclose all analysis results and achieve information transparency.*
